# Glucose tolerance and insulin resistance/sensitivity associate with retinal layer characteristics: the LIFE-Adult-Study

**DOI:** 10.1007/s00125-024-06093-9

**Published:** 2024-03-02

**Authors:** Franziska G. Rauscher, Tobias Elze, Mike Francke, M. Elena Martinez-Perez, Yangjiani Li, Kerstin Wirkner, Anke Tönjes, Christoph Engel, Joachim Thiery, Matthias Blüher, Michael Stumvoll, Toralf Kirsten, Markus Loeffler, Thomas Ebert, Mengyu Wang

**Affiliations:** 1 Leipzig Research Centre for Civilization Diseases (LIFE), Leipzig University, Leipzig, Germany; 2 Institute for Medical Informatics, Statistics, and Epidemiology (IMISE), Leipzig University, Leipzig, Germany; 3 Schepens Eye Research Institute of Mass Eye and Ear, Department of Ophthalmology, Harvard Medical School, Boston, MA USA; 4 Instituto de Investigaciones en Matemáticas Aplicadas y en Sistemas, Universidad Nacional Autónoma de México, Ciudad de México, Mexico; 5 Medical Department III – Endocrinology, Nephrology, Rheumatology, University of Leipzig Medical Center, Leipzig, Germany; 6 Institute of Laboratory Medicine, Clinical Chemistry and Molecular Diagnostics, Leipzig University, Leipzig, Germany; 7 Helmholtz Institute for Metabolic, Obesity and Vascular Research (HI-MAG) of the Helmholtz Zentrum München at the University of Leipzig and University Hospital Leipzig, Leipzig, Germany; 8 Department of Medical Data Science, University of Leipzig Medical Center, Leipzig, Germany

**Keywords:** Diabetes, Early Treatment Diabetic Retinopathy Study, ETDRS, Glucose tolerance, Insulin resistance, Insulin sensitivity, Optical coherence tomography, Population-based, Prediabetes, Retinal layer thickness

## Abstract

**Aims/hypothesis:**

As the prevalence of insulin resistance and glucose intolerance is increasing throughout the world, diabetes-induced eye diseases are a global health burden. We aim to identify distinct optical bands which are closely related to insulin and glucose metabolism, using non-invasive, high-resolution spectral domain optical coherence tomography (SD-OCT) in a large, population-based dataset.

**Methods:**

The LIFE-Adult-Study randomly selected 10,000 participants from the population registry of Leipzig, Germany. Cross-sectional, standardised phenotyping included the assessment of various metabolic risk markers and ocular imaging, such as SD-OCT-derived thicknesses of ten optical bands of the retina. Global and Early Treatment Diabetic Retinopathy Study (ETDRS) subfield-specific optical retinal layer thicknesses were investigated in 7384 healthy eyes of 7384 participants from the LIFE-Adult-Study stratified by normal glucose tolerance, prediabetes (impaired fasting glucose and/or impaired glucose tolerance and/or HbA_1c_ 5.7–6.4% [39–47 mmol/mol]) and diabetes. The association of optical retinal band characteristics with different indices of glucose tolerance (e.g. fasting glucose, area under the glucose curve), insulin resistance (e.g. HOMA2-IR, triglyceride glucose index), or insulin sensitivity (e.g. estimated glucose disposal rate [eGDR], Stumvoll metabolic clearance rate) was determined using multivariable linear regression analyses for the individual markers adjusted for age, sex and refraction. Various sensitivity analyses were performed to validate the observed findings.

**Results:**

In the study cohort, nine out of ten optical bands of the retina showed significant sex- and glucose tolerance-dependent differences in band thicknesses. Multivariable linear regression analyses revealed a significant, independent, and inverse association between markers of glucose intolerance and insulin resistance (e.g. HOMA2-IR) with the thickness of the optical bands representing the anatomical retinal outer nuclear layer (ONL, standardised *β*=−0.096; *p*<0.001 for HOMA2-IR) and myoid zone (MZ; *β*=−0.096; *p*<0.001 for HOMA2-IR) of the photoreceptors. Conversely, markers of insulin sensitivity (e.g. eGDR) positively and independently associated with ONL (*β*=0.090; *p*<0.001 for eGDR) and MZ (*β*=0.133; *p*<0.001 for eGDR) band thicknesses. These global associations were confirmed in ETDRS subfield-specific analyses. Sensitivity analyses further validated our findings when physical activity, neuroanatomical cell/tissue types and ETDRS subfield categories were investigated after stratifying the cohort by glucose homeostasis.

**Conclusions/interpretation:**

An impaired glucose homeostasis associates with a thinning of the optical bands of retinal ONL and photoreceptor MZ. Changes in ONL and MZ thicknesses might predict early metabolic retinal alterations in diabetes.

**Graphical Abstract:**

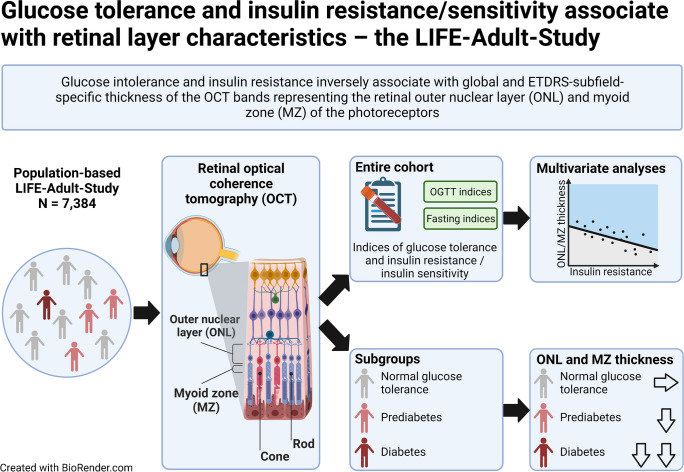

**Supplementary Information:**

The online version contains peer-reviewed but unedited supplementary material available at 10.1007/s00125-024-06093-9.


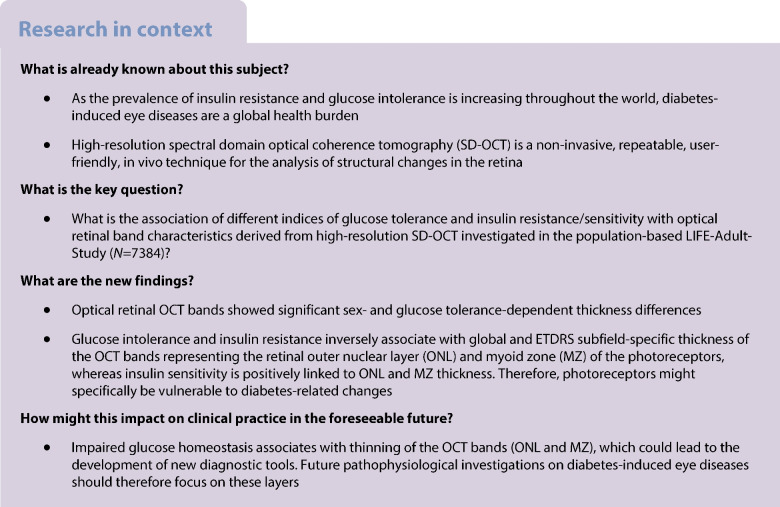


## Introduction

Diabetes mellitus is a global health burden and the increasing prevalence throughout the world contributes to an unacceptably large number of disability-adjusted life-years [1]. Diabetic retinopathy is a severe and feared complication and one of the most common causes of blindness or severe vision impairment [2], and poor eye health and impaired vision have a significant, negative effect on quality of life in affected individuals [3].

**Fig. 1:**
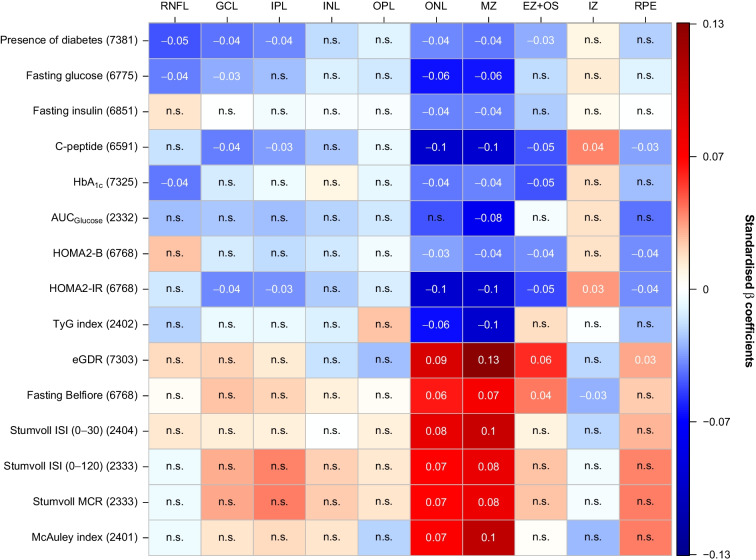
Heatmap of standardised *β* coefficients for all investigated markers of glucose homeostasis and the ten different retinal band thicknesses. Separate multivariable linear regression analyses were carried out for each of the markers (independent variable) and the respective retinal band thickness (dependent variable). All multivariable models were adjusted for age, sex and refraction. The false discovery rate method was applied to correct *p* values for multiple comparisons. For all multivariable models, strength as assessed by standardised *β*, as well as the direction, of the associations are colour-coded. Thus, positive (in red/warmer colours) and negative (in blue/cooler colours) associations are shaded based on the respective standardised *β* coefficients. The exact standardised *β* coefficient is given for all significant models with *p*<0.05. If the respective linear regression model did not show an overall significance (indicating that the standardised *β* is not valid for this association), no exact standardised *β* coefficients are depicted. Number of participants included in all multivariate models for the respective marker of glucose homeostasis are provided. n.s., not significant; Stumvoll ISI, Stumvoll insulin sensitivity index

Spectral domain optical coherence tomography (SD-OCT) is a non-invasive, repeatable, user-friendly, in vivo technique [4] for the analysis of structural changes in the retina [5]. Importantly, SD-OCT-based quantification of retinal layer thickness could be an easy, affordable and unbiased tool to detect retinal abnormalities, including neurodegenerative changes in people with diabetes [5]. A standardised nomenclature system for SD-OCT has been developed, thereby harmonising the SD-OCT outputs throughout different OCT instruments and diverse populations [6]. In more detail, several anatomical landmarks can be detected by SD-OCT bands, which correspond to some, but not all, anatomical retinal layers identified by distinct hyperreflective and hyporeflective features in SD-OCT [6, 7]. SD-OCT bands technically represent optical properties of the neuronal/retinal tissue and specifically the subcellular components. Furthermore, optical OCT bands (herein after referred to as bands) within the retina (i.e. from internal limiting membrane [ILM] to the external limiting membrane [ELM]) correspond well to the anatomical layers of the retina with their different optical properties. Thus, the retinal nerve fibre layer (RNFL) contains various cell types, such as microglial cells, astrocytes, parts of Müller cells, and the axons from the ganglion cells and blood vessels. The ganglion cell layer (GCL) comprises the somata of the ganglion cells, whereas the synaptic terminals between bipolar, ganglion and amacrine cells are located in the inner plexiform layer (IPL). Furthermore, the IPL contains microglial cells and the proximal cell processes of Müller cells. The inner nuclear layer (INL) consists of the cell somata of bipolar, amacrine and Müller cells, respectively. The outer plexiform layer (OPL) comprises synaptic connections between the photoreceptors (cone pedicles and rod spherules), bipolar cells and horizontal cells and contains microglia cells. Furthermore, the Henle's fibre layer (HFL) in the central macular part contains the axonal terminal cell processes of cones and rods, as well as the centrifugal oriented distal processes of Müller cells. The outer nuclear layer (ONL) predominantly consists of cone and rod somata. Additional optical bands appear in the subretinal space (distal from the ELM) representing parts of the photoreceptors (myoid zone [MZ], ellipsoid zone [EZ], outer segment [OS]), the interdigitation zone (IZ; anatomically the interacting area of the outermost part of the outer-photoreceptor segments and the microvilli from the retinal pigment epithelium [RPE]) and the basal part of the RPE cells and the Bruch's membrane [6] (representative examples from the study cohort are shown in electronic supplementary material [ESM] Fig. 1).

The effect of diabetes status, glucose tolerance and insulin resistance per se (i.e. in the absence of diabetic retinopathy) on the different retinal layer characteristics have not, to date, been investigated in large population-based studies enabling the adjustment for age, sex, refraction and other clinically relevant covariates.

Pathophysiologically, the first evidence of an effect of diabetes on the thickness of retinal layers is based on animal experiments using diabetic mouse models where reductions in the thickness of the IPL and INL were found [8]. Human studies then validated these findings and reported thinning of distinct optical retinal OCT bands in small studies of patients with type 2 diabetes [9, 10], as well as in type 1 diabetes [11]. In an elegant study by De Clerck and co-workers, individuals with type 2 diabetes and prediabetes showed a thinning of the macula within a 3 mm area around the fovea analysed in five Early Treatment Diabetic Retinopathy Study (ETDRS) subfields [12], which suggests that glucose metabolism systemically affects retinal layer characteristics. However, previous studies investigating layer-specific characteristics in participants with impaired glucose metabolism show the following limitations: they (1) included cohorts of smaller sample size (mostly *N*<150); (2) have analysed only distinct bands, but not the entire retinal spectrum comprising ten SD-OCT-derived bands; (3) have investigated only distinct patient groups, i.e. control participants compared with diabetic patient groups, but not continuous measures of glucose metabolism; (4) did not include a wide range of anthropometric, biochemical, cardiometabolic markers, and other patient-level data; and (5) did not use thoroughly adjusted multivariable models to investigate independent predictors of the respective retinal bands.

We, therefore, have investigated a large panel of well-established markers of glucose metabolism and insulin resistance/sensitivity using static (e.g. fasting glucose and insulin-derived indices, such as HOMA indices), but also dynamic, OGTT-based (e.g. area under the glucose curve [AUC_Glucose_], Stumvoll) indices and their associations with SD-OCT band thicknesses of ten different optical retinal bands measured by SD-OCT in the large (*N*=7384 participants), unselected, and deeply phenotyped, population-based LIFE-Adult-Study in Germany.

Our hypothesis is that glucose metabolism and insulin resistance/sensitivity are associated with a specific association pattern with the SD-OCT band thicknesses, independent of diabetes status and glycaemic control and similar to other cardiometabolic diseases, e.g. reduced RNFL thickness in individuals with impaired renal function [13]. We aim to identify the optical retinal bands, as visible on SD-OCT, which show strongest associations with insulin and glucose metabolism. As the prevalence of diabetes and insulin resistance is increasing throughout the world [1], this is of particular interest before implementing layer-specific retinal SD-OCT analyses into clinics and research. Furthermore, altered OCT-derived retinal band thicknesses could potentially identify patients at risk prior to clinically relevant and fundus photograph-identifiable retinopathy.

## Methods

### Participants

This analysis is part of the population-based LIFE-Adult-Study conducted by the Leipzig Research Centre for Civilization Diseases at Leipzig University between August 2011 and November 2014 [14, 15]. The LIFE-Adult-Study has recruited about 10,000 randomly selected participants from the population registry of the just over half a million inhabitants of Leipzig in an age- and sex-stratified fashion, thereby covering a main age range from 40 to 79 years. The overall aim of the LIFE-Adult-Study is to investigate prevalences and predictors of major diseases of civilisation, with primary focus on metabolic and vascular diseases, cardiac function, cognitive impairment, brain function, depression, sleep disorders and vigilance dysregulation, retinal and optic nerve degeneration, and allergies [14]. The study was approved by the Ethical Committee at the Medical Faculty of Leipzig University (approval number: 263-2009-14122009) and adheres to the Declaration of Helsinki and all federal and state laws. Prior to inclusion, informed written consent was obtained from all participants.

### Data collection/inclusion and exclusion criteria

A detailed description of inclusion and exclusion criteria, as well as the cardiometabolic phenotyping, of the LIFE-Adult-Study can be found in the ESM Methods and ESM Fig. 2.

Ophthalmological and clinical phenotyping, as well as data collection, at baseline have been described elsewhere [13, 14, 16, 17].

Briefly, SD-OCT imaging (Spectralis, Heidelberg Engineering, Heidelberg, Germany) of the macula was performed using a visual angle of 20° × 20° by 97 B-scans with a distance of 60 µm, respectively (acquisition software: HEYEX software version 5.4.7.0, Heidelberg Engineering). All SD-OCT measurements took place in a dark room 3 min after the lights were switched off following visual acuity measurement. While the light level was the same for each participant, no quantitative control of the illumination level was performed. The Advised Protocol for OCT Study Terminology and Elements (APOSTEL) 2.0 checklist for our OCT measurements can be found in the ESM. Examiners were blinded to participants' glucose homeostasis and diabetes status.

### Post-acquisition retinal SD-OCT image analysis

Retinal optical bands were automatically segmented by HEYEX software version 6.16.8 (Heidelberg Engineering) yielding ten different retinal bands, i.e. RNFL, GCL, IPL, INL, OPL, ONL (which includes thickness of ELM as this layer is segmented by HEYEX software at the inferior border of the bright band [ELM]), MZ, EZ and OS combined (named EZ+OS; segmentation by HEYEX software of this section consists of a bright [EZ] and a dark [OS] band), IZ and RPE. Automatic segmentation was adequate as verified by visual inspection of random samples, and no segmentation adjustment was made. Importantly, the visibility of the HFL containing photoreceptor axonal cell processes and interleaved Müller glial processes in OCT is angle-dependent [18, 19]. This may lead to a redefinition of the nomenclature for OCT layers; indeed, although tomographically the HFL seems to be part of the ONL in OCT, it should be anatomically considered as part of the OPL, as described by Mrejen and co-workers [20]. Thus, HFL is part of the ONL in all of our SD-OCT-derived analyses. As relevant retinal cell types span several retinal layers, different bands were grouped to represent relevant cell types, i.e. photoreceptors, ganglion cells and bands containing the intrinsic retinal vasculature. For this purpose, photoreceptor-containing bands were defined as the combined thickness of OPL, ONL including HFL, MZ, EZ+OS, as well as IZ. Furthermore, ganglion cell-containing bands were defined as RNFL, GCL and IPL. Moreover, bands containing the intrinsic retinal vasculature were summarised by all bands from the ILM to ONL, but not including ONL.

Layer data were averaged into a global mean for each retinal band, as well as into ETDRS subfields according to the ETDRS manual of operations [21]. The ETDRS grid divides the retina into nine large subfields defined by three rings: a central foveal ring with 1 mm diameter (A1), an inner macular ring (parafovea; A2–A5) with 3 mm diameter, and an outer macular ring (perifovea; A6–A9) with 6 mm diameter. Each ring is segmented into four quadrants (superior, inferior, nasal, temporal). Thickness measurement in each of the nine subfields is estimated by averaging the thickness measurements within this subfield for the respective retinal band investigated.

### Anthropometric and biochemical markers and markers of glucose homeostasis

At baseline, past medical history, routine anthropometric measurements and fasting blood samples were collected (ESM Methods). Sex of all participants was self-reported. In a subcohort, a standardised 75 g OGTT was conducted [14]. Routine blood variables were measured in a certified laboratory by standard methods.

A detailed description of the assessment of different markers of glucose homeostasis, insulin resistance and insulin sensitivity can be found in ESM Table 1. Briefly, AUC_Glucose_ was calculated according to the trapezoidal rule [22]. HOMA2 beta cell function (HOMA2-B) and insulin resistance (HOMA2-IR) were determined by HOMA2 Calculator software version 2.2.3 (University of Oxford, UK) using C-peptide levels. Triglyceride glucose (TyG) index, estimated glucose disposal rate (eGDR), fasting Belfiore index, Stumvoll insulin sensitivity index, Stumvoll metabolic clearance rate (MCR) and McAuley index were calculated with standard equations depicted in ESM Table 1. Diabetes, prediabetes (presence of impaired fasting glucose and/or impaired glucose tolerance and/or HbA1c between 5.7–6.4% [39–47 mmol/mol]), as well as normal glucose tolerance (NGT), were defined according to the ADA definition [23]. Chronic kidney disease (CKD) status was defined as a urinary albumin/creatinine ratio ≥ 30 mg/g and/or a decreased eGFR <60 ml/min per 1.73 m^2^ [24].

### Statistical analysis

A full description of the statistical analyses can be found in the ESM Methods. Briefly, all statistical analyses were performed in R environment using version 4.0 (R Foundation for Statistical Computing, Vienna, Austria). Group-wise comparisons were calculated using ANOVA and/or unpaired student *t* test (for continuous variables) or *χ*^2^ test (for categorical variables).

Multivariable linear regression analyses were calculated for each marker of glucose homeostasis and retinal band thicknesses adjusted for age, sex and refraction, respectively. To further identify whether a marker of glucose homeostasis is substantially related to the global thickness of a specific retinal band, the increase of Bayesian information criterion (ΔBIC) > +2 was used.

**Fig. 2:**
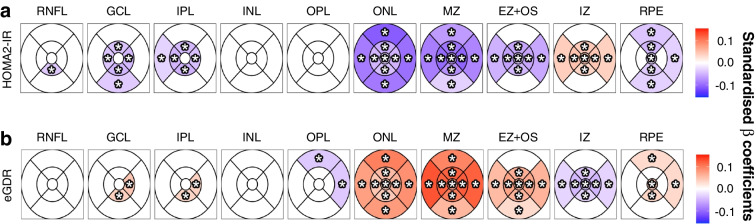
Subfield-specific associations between key markers of insulin resistance and insulin sensitivity. Retinal thickness of ten macular bands averaged within the commonly used ETDRS subfields were investigated for HOMA2-IR (marker for insulin resistance) (**a**), and eGDR (marker for insulin sensitivity) in the entire cohort (*N*=7384) (**b**). Standardised *β* coefficients are colour-coded depicting the strength and direction of the association. Thus, positive (in red/warmer colours) and negative (in blue/cooler colours) associations are shaded based on the respective standardised *β* coefficients. Asterisks denote statistically significant associations after adjustment for multiple comparisons by the false discovery rate method. In all non-coloured (i.e. white) sectors, no statistical significance was observed

We further associated ETDRS-based average subfield thickness for each of the optical macular bands with key markers of insulin resistance and sensitivity with subfield-specific linear regression models adjusted for age, sex and refraction (Fig. 2).

Different sensitivity analyses were carried out as depicted in the ESM Methods.

A *p* value of <0.05 (two-sided) was considered statistically significant in all analyses.

## Results

### Baseline characteristics of the current study population (*N*=7384)

Baseline characteristics of the entire cohort, as well as stratified by glucose tolerance status, are shown in Table 1. A total of 7384 (3933 female and 3451 male) eyes from the population-based LIFE-Adult-Study were selected for the present analysis according to our criteria described in the Methods section. Mean age ± SD of the total population was 56.2±12.2 years (Table 1). A total of 4209 participants showed an NGT according to the ADA definition [23], whereas 2226 individuals were classified as having prediabetes and 949 patients had a diagnosis of diabetes (Table 1). Individuals with prediabetes and diabetes had a higher prevalence of hypertension and current statin treatment, whereas smoking prevalence was reduced in diabetes (Table 1, *p*<0.05). Furthermore, NGT participants had a more beneficial metabolic profile compared with prediabetic and diabetic individuals, including markers of obesity (i.e. BMI), BP, glucose homeostasis (i.e. fasting glucose, HbA_1c_), dyslipidaemia, and inflammation (high-sensitivity C-reactive protein) (all *p*<0.05; Table 1).

**Table 1:** Baseline characteristics of the entire study population, as well as subgroups stratified by glucose homeostasis (*N*=7384)

Characteristic	Entire cohort	Normal glucose tolerance	Prediabetes	Diabetes	*p* between subgroups
Total *N*	7384	4209	2226	949	–
Age, years	56.2±12.2	52.4±12.0	59.9±10.6	64.6±9.1	<0.001
Sex, *n* female/male participants	3933/3451	2559/1650	951/1275	423/526	<0.001
Smoker, *n* (%)	1623 (22.0)	1007 (23.9)	453 (20.4)	163 (17.2)	<0.001
Hypertension, *n* (%)	3522 (47.7)	1404 (33.4)	1349 (60.6)	769 (81.0)	0.001
Statin therapy, *n* (%)	842 (11.4)	246 (5.8)	278 (12.5)	318 (33.5)	<0.001
CKD, *n* (%)	1048 (14.2)	374 (8.9)	359 (16.1)	315 (33.2)	<0.001
BMI, kg/m^2^	27.3±4.9	25.8±4.3	28.5±4.9	30.6±5.1	<0.001
SBP, mmHg	127.9±16.7	125.0±16.0	131.3±16.4	132.9±17.5	<0.001
DBP, mmHg	75.4±9.8	74.7±9.6	76.8±9.9	74.7±9.9	0.001
Fasting glucose, mmol/l	5.6±1.1	5.1±0.3	5.9±0.4	7.6±2.1	<0.001
HbA_1c_, mmol/mol	35.3±6.1	32.9±3.1	35.8±3.8	44.6±10.4	<0.001
HbA_1c_, %	5.4±0.6	5.2±0.3	5.4±0.4	6.2±1.0	<0.001
Total cholesterol, mmol/l	5.6±1.1	5.5±1.0	5.8±1.1	5.4±1.1	0.798
HDL-cholesterol, mmol/l	1.6±0.5	1.7±0.5	1.6±0.5	1.4±0.4	<0.001
LDL-cholesterol, mmol/l	3.5±1.0	3.4±0.9	3.7±1.0	3.3±1.0	0.055
TG, mmol/l	1.4±1.1	1.2±0.8	1.6±1.2	1.8±1.4	<0.001
hsCRP, mg/l	2.8±5.7	2.3±3.9	3.2±6.3	4.0±9.6	<0.001

Data are expressed as mean ± SD or *n* (%)

Overall *p* values were assessed by ANOVA or *χ*^2^ test and corrected for multiple testing based on the false discovery rate method, respectively

DBP, diastolic BP; hsCRP, high-sensitivity C-reactive protein; SBP, systolic BP; TG, triglycerides

### Global retinal layer thicknesses: sex- and glucose homeostasis-stratified analyses

Mean averaged thicknesses for all ten investigated retinal bands stratified by sex (ESM Table 2) and glucose homeostasis (ESM Table 2, ESM Fig. 3) are given. Female participants had significantly thicker RNFL and MZ compared with male participants, whereas all other bands were significantly thinner in female individuals, except OPL (all *p*<0.05; ESM Table 2). For both sexes, participants with diabetes showed thinner RNFL, GCL, IPL, INL, ONL, MZ, EZ+OS compared with non-diabetic individuals (all *p*<0.05, sex-stratified results in ESM Table 2; combined data in ESM Fig. 3). In contrast, participants with diabetes had thicker IZ compared with non-diabetic individuals (*p*<0.05, ESM Table 2, ESM Fig. 3). Results remained virtually the same when analyses were stratified by physical activity categories (ESM Table 3). When different retinal bands were grouped to represent relevant retinal cell types, i.e. ganglion cells, photoreceptor cells and the intrinsic retinal vasculature, thickness of all of these three cell/tissue types were also significantly reduced in individuals with diabetes compared to those with NGT (all *p*<0.05; ESM Table 4).

When individuals with type 1 diabetes were compared to those with type 2 diabetes, none of the ten investigated retinal bands significantly differed in terms of thickness between both types of diabetes (ESM Table 5).

### Associations between global retinal layer thickness of ten retinal bands and markers of glucose metabolism and insulin resistance

We next sought to identify distinct retinal bands that are associated with markers of glucose tolerance and in particular insulin sensitivity/resistance. The overall pattern suggested that markers of glucose tolerance (e.g. fasting glucose, AUC_Glucose_ as assessed by the trapezoidal rule [22]) and insulin resistance (e.g. HOMA2-IR, TyG index) were mostly negatively, whereas different indices of insulin sensitivity (e.g. eGDR, Stumvoll MCR) were mostly positively, related to global retinal thicknesses of the different retinal bands, referring to a global retinal thinning when adverse glucose homeostasis is present. In contrast, only the IZ band was inversely correlated to the investigated markers of glucose metabolism (Table 2 and Fig. 1).

**Table 2:** Multivariate linear regression models and Bayesian information criterion differences on the association between glucose homeostasis and retinal layer thicknesses

Variable		RNFL	GCL	IPL	INL	OPL	ONL	MZ	EZ+OS	IZ	RPE
Conventional markers of glucose tolerance											
Presence of diabetes	*β*/*p*	−0.048/<0.001	−0.041/0.001	−0.037/0.003	−0.018/0.141	−0.01/0.462	−0.037/0.003	−0.04/0.001	−0.03/0.013	0.009/0.462	−0.021/0.108
	ΔBIC	8.000	4.610	1.280	−6.390	−8.280	1.300	3.880	−1.770	−8.370	−5.750
Fasting glucose	*β*/*p*	−0.039/0.006	−0.029/0.036	−0.025/0.08	−0.011/0.402	−0.014/0.378	−0.062/<0.001	−0.06/<0.001	−0.018/0.221	0.013/0.378	−0.01/0.459
	ΔBIC	0.833	−2.840	−4.610	−7.990	−7.610	15.500	16.100	−6.560	−7.760	−8.270
Fasting insulin	*β*/*p*	0.015/0.52	0/0.989	−0.005/0.989	−0.003/0.989	−0.003/0.989	−0.038/0.005	−0.039/0.005	−0.022/0.153	0.005/0.989	0/0.989
	ΔBIC	−7.245	−8.830	−8.630	−8.770	−8.770	2.080	2.760	−4.840	−8.620	−8.830
C-peptide	*β*/*p*	−0.017/0.2	−0.039/0.003	−0.033/0.01	−0.023/0.07	−0.006/0.626	−0.098/<0.001	−0.095/<0.001	−0.047/<0.001	0.038/0.004	−0.032/0.018
	ΔBIC	−6.994	2.000	−1.300	−5.150	−8.560	54.570	55.260	6.880	0.590	−2.560
HbA_1c_	*β*/*p*	−0.04/0.002	−0.013/0.357	−0.005/0.636	0.008/0.588	−0.008/0.588	−0.04/0.002	−0.038/0.002	−0.048/<0.001	0.017/0.256	−0.025/0.075
	ΔBIC	2.839	−7.580	−8.670	−8.380	−8.500	2.980	2.080	8.800	−6.860	−4.570
AUC_Glucose_	*β*/*p*	−0.026/0.437	−0.023/0.437	−0.026/0.437	−0.021/0.468	−0.014/0.578	−0.047/0.14	−0.076/0.003	−0.004/0.865	0.016/0.574	−0.041/0.204
	ΔBIC	−6.283	−6.490	−6.320	−6.790	−7.340	−2.920	5.100	−7.730	−7.210	−4.250
Markers of insulin resistance											
HOMA2-B	*β*/*p*	0.025/0.069	−0.013/0.289	−0.018/0.196	−0.014/0.288	−0.005/0.672	−0.032/0.015	−0.039/0.006	−0.036/0.008	0.016/0.229	−0.036/0.01
	ΔBIC	−4.357	−7.550	−6.370	−7.380	−8.640	−1.310	2.990	1.170	−6.850	0.050
HOMA2-IR	*β*/*p*	−0.014/0.268	−0.037/0.004	−0.035/0.007	−0.023/0.074	−0.012/0.362	−0.096/<0.001	−0.096/<0.001	−0.05/<0.001	0.034/0.007	−0.036/0.007
	ΔBIC	−7.443	1.290	−0.590	−5.270	−7.990	53.310	57.280	8.990	−0.970	−0.880
TyG index	*β*/*p*	−0.02/0.645	−0.007/0.809	−0.007/0.809	−0.011/0.809	0.024/0.644	−0.063/0.011	−0.097/<0.001	0.017/0.654	−0.001/0.959	−0.025/0.644
	ΔBIC	−6.803	−7.660	−7.660	−7.520	−6.500	1.680	14.980	−7.050	−7.780	−6.380
Markers of insulin sensitivity											
eGDR	*β*/*p*	0.018/0.179	0.021/0.144	0.013/0.289	−0.016/0.231	−0.026/0.094	0.09/<0.001	0.133/<0.001	0.055/<0.001	−0.019/0.173	0.031/0.045
	ΔBIC	−6.755	−5.950	−7.770	−7.310	−4.940	42.380	110.980	12.170	−6.490	−3.310
Fasting Belfiore	*β*/*p*	0.004/0.784	0.025/0.06	0.021/0.116	0.011/0.447	0.003/0.784	0.06/<0.001	0.072/<0.001	0.041/0.001	−0.028/0.044	0.023/0.113
	ΔBIC	−8.720	−4.120	−5.770	−7.970	−8.740	16.120	29.430	3.630	−3.180	−5.490
Stumvoll ISI (0–30)	*β*/*p*	0.014/0.675	0.012/0.675	0.011/0.675	0/0.983	0.011/0.675	0.081/0.001	0.099/<0.001	0.01/0.675	−0.019/0.675	0.027/0.675
	ΔBIC	−7.330	−7.430	−7.520	−7.780	−7.510	7.330	15.810	−7.520	−6.900	−6.160
Stumvoll ISI (0–120)	*β*/*p*	−0.006/0.823	0.03/0.276	0.038/0.183	0.021/0.433	0.014/0.643	0.067/0.006	0.08/0.001	0.024/0.376	−0.005/0.823	0.038/0.183
	ΔBIC	−7.668	−5.550	−4.440	−6.690	−7.330	2.680	7.650	−6.280	−7.700	−4.540
Stumvoll MCR	*β*/*p*	−0.005/0.843	0.031/0.255	0.039/0.165	0.023/0.381	0.015/0.61	0.066/0.007	0.079/0.001	0.025/0.356	−0.004/0.843	0.039/0.165
	ΔBIC	−7.687	−5.430	−4.270	−6.520	−7.270	2.510	7.070	−6.200	−7.720	−4.370
McAuley index	*β*/*p*	−0.006/0.847	0.015/0.584	0.018/0.584	0.015/0.584	−0.02/0.584	0.068/0.004	0.105/<0.001	0.003/0.889	−0.026/0.479	0.039/0.21
	ΔBIC	−7.692	−7.200	−6.970	−7.250	−6.890	3.480	19.820	−7.760	−6.070	−4.320

For each of the ten investigated retinal bands, a linear regression model was calculated with age, sex and refraction, as well as the respective marker of glucose homeostasis, as regressors

Standardised *β* coefficients and corresponding *p* values (corrected for multiple testing based on the false discovery rate method) for the respective marker of glucose homeostasis are depicted

As a validation analysis, ΔBIC was calculated for the association of the respective marker of glucose homeostasis and retinal layer thicknesses. For this purpose, two different linear regression models were calculated with age, sex, refraction, and the respective glucose marker as regressors (model A), as well as an additional model comprising age, sex and refraction only (model B). The BIC difference (ΔBIC) was calculated by ΔBIC = BIC_model B_ − BIC_model A_. ΔBIC values are depicted and a ΔBIC >2 was regarded as statistically relevant

Stumvoll ISI, Stumvoll insulin sensitivity index

Linear regression analyses revealed significant and independent associations with almost all investigated indices of glucose homeostasis especially for the ONL and MZ bands (*p*<0.05, Table 2 and Fig. 1). For an increase in 1.0 unit of HOMA2-IR, there was a reduction in global ONL and MZ thickness of 0.69 µm and 0.06 µm, respectively. Furthermore, each increase of 1.0 in the insulin sensitivity marker eGDR resulted in an increased thickness in ONL and MZ of 0.39 µm and 0.05 µm, respectively.

In contrast, the IZ was the only band that showed inverse, significant associations for some of the markers (C-peptide, HOMA2-IR, fasting Belfiore) (*p*<0.05, Table 2 and Fig. 1). As a validation of the multivariable analyses, ΔBIC >2 was evident in the bands ONL and MZ for most of the investigated markers of glucose homeostasis (Table 2).

Based on the number of significant associations and the relative importance according to their ΔBIC values (Table 2), HOMA2-IR and eGDR stood out as the most relevant markers and, therefore, were selected for further ETDRS subfield-specific retinal analyses.

### Associations between different ETDRS subfield-specific retinal layer thicknesses and insulin resistance and insulin sensitivity

Overall pattern of the associations between ETDRS subfield-specific retinal band thicknesses and the insulin resistance-related HOMA2-IR (Fig. 2a) were inverse compared with the insulin sensitivity marker eGDR (Fig. 2b). However, statistically significant sectors were equally distributed across all retinal bands and all ETDRS subfields (Fig. 2). Thus, all subfields of the bands ONL and MZ were independently and negatively associated with HOMA2-IR (Fig. 2a), whereas eGDR remained a positive and independent predictor of all subfields in ONL and MZ in the entire cohort. In several other retinal bands, distinct subfields were related to both markers in the same manner. In contrast, the central inner subfields of IZ were inversely related to HOMA2-IR and eGDR as compared with all other retinal bands (Fig. 2).

We further compared retinal band thicknesses between patients with diabetes, prediabetes and NGT stratified by retinal subfields, i.e. in perifoveal-only (i.e. subfields A6, A7, A8 and A9 from the outer ring of the ETDRS grid), in parafoveal-only (i.e. subfields A2, A3, A4 and A5 from the inner ring of the ETDRS grid), and in the central foveal subfield A1-only (ESM Table 6). For ONL and MZ, patients with diabetes had significantly lower band thicknesses compared with NGT control individuals in perifoveal and parafoveal subfields (ESM Table 6).

### Sensitivity analyses—NGT, prediabetes, diabetes subgroups

We carried out different sensitivity analyses by stratifying our cohort by glucose homeostasis. Thus, global retinal band thicknesses were analysed in NGT individuals only (ESM Fig. 4), in prediabetes participants only (ESM Fig. 5), as well as in individuals with diabetes (ESM Fig. 6) separately. Associations between glucose homeostasis markers and global retinal band thicknesses remained virtually the same for the glucose homeostasis-responsible bands ONL and MZ in NGT and prediabetes individuals (ESM Figs 1, 2). In contrast, participants with diabetes showed attenuated associations (ESM Fig. 6). In ETDRS subfield-specific sensitivity analyses stratified by glucose homeostasis subgroups, a similar pattern was detectable for ONL and MZ in all subgroups for HOMA2-IR (ESM Fig. 7a) and eGDR (ESM Fig. 7b).

## Discussion

In our large dataset comprising 7384 deeply phenotyped participants, we demonstrate that individuals with diabetes showed thinner global retinal layer thickness in most bands compared with non-diabetic individuals in both sexes. After multivariable adjustment, glucose tolerance, insulin resistance and insulin sensitivity are significantly and independently associated with global thickness of two distinct retinal bands, i.e. ONL and MZ. Thus, markers of insulin sensitivity (e.g. eGDR) are positively related to ONL and MZ thickness, whereas an impaired glucose tolerance (e.g. HbA_1c_) and insulin resistance (e.g. HOMA2-IR) are inversely related to ONL and MZ thickness. Moreover, we provide sex-stratified normative data for macular band thicknesses specifically for individuals with diabetes, prediabetes and NGT.

High-resolution SD-OCT is a well-established tool to link retinal changes with intraocular (e.g. glaucoma [25, 26]) and extraocular (e.g. CKD [13] and dyslipidaemia [13]) diseases. SD-OCT can be used for the investigation of diabetes-induced eye diseases [27, 28]. However, the effects of a diabetes diagnosis, as well as glucose homeostasis and insulin resistance/sensitivity, on the characteristics of a large number of retinal bands at distinct retinal locations over a large macular area have not, to date, been investigated. Our data indicate that systemic insulin resistance and impaired glucose homeostasis are linked to thinner global and sector-specific ONL and MZ thickness, independent of a diabetes diagnosis. Furthermore, insulin resistance and sensitivity are early markers of metabolic retinal alterations even prior to clinically evident eye diseases and show stronger associations with ONL and MZ thickness compared with hyperglycaemia.

It is interesting to note in this context that hyperglycaemia has been demonstrated as a major pathogenetic component in early diabetic retinopathy [2]. However, we aimed to investigate whether other markers of glucose homeostasis in addition to hyperglycaemia (i.e. insulin resistance and insulin sensitivity) are associated with early metabolic retinal alterations prior to the development of diabetic retinopathy. Based on our results, functional studies are needed to further identify the pathomechanisms by which insulin resistance/sensitivity impairs ONL and MZ layer physiology contributing to associated visual complications, for instance loss of chromatic sensitivity [29]. Both optical bands (i.e. ONL and MZ) represent parts of the photoreceptor cells: the somatic area with the nucleus (ONL) and the myoid part with important metabolic components, such as the smooth and rough endoplasmatic reticulum, Golgi apparatus, and various filaments.

Mechanistically, if insulin resistance/sensitivity associates with differential retinal layer characteristics, a functional insulin signalling cascade would be required. It is interesting to note in this context that the insulin receptor is expressed in several inner and outer retinal layers, including ONL and MZ [30, 31]. Importantly, ONL and MZ comprise different cell types, e.g. photoreceptor cells, responsible for photon signal transmission [32], as well as Müller cells forming junctional complexes with photoreceptors (i.e. the ELM) [33]. Photoreceptor cells are the most metabolically active cell types throughout the retina crucially depending on glucose metabolism [34]. Furthermore, glucose metabolism-relevant receptors (e.g. insulin receptor), the post-insulin receptor signalling cascade (e.g. insulin receptor substrate-1) and glucose transporters have been shown to be expressed on Müller cells and photoreceptor cells [30, 34]. To incorporate principles of retinal neuroanatomy, vascular biology and OCT signal generation in our analysis, we have grouped retinal band thicknesses representing relevant retinal cell types of ganglion cells, the intrinsic retinal vasculature, as well as photoreceptor cells. Importantly, for all of these cell/tissue types, we show reduced thickness in patients with diabetes compared to NGT individuals, strongly supporting our findings in retinal band-specific analyses.

Taking our data, and previous data, into consideration, these retinal layers, i.e. ONL and MZ, might be susceptible to changes in insulin resistance/sensitivity and glucose homeostasis due to their functional insulin signalling cascade. We cannot exclude that the retinal band thinning observed in our study might be a physiological response to protect from diabetes-induced oedema, hinted at by the very low diabetic macular oedema prevalence in the Joslin 50-Year Medalist Study [35]. Clearly, the reasons for the observed decreased thicknesses of ONL and MZ in insulin resistance need to be investigated in future pathophysiological experiments on an ultrastructural level and it needs to be elucidated whether hyperglycaemia and insulin resistance show differential effects on retinal layer characteristics. Interestingly, some but not all studies have demonstrated that structural changes in the retina, including a reduction in ONL thickness, occur in several rodent models of diabetes [36]. It is, therefore, tempting to speculate that insulin resistance can either directly or indirectly (for instance through increased oxidative stress and/or inflammation) impair photoreceptor cell shape and/or cell death [36]. Notably, physical activity as an important lifestyle contributor that impacts insulin sensitivity does not appear to be a major factor confounding our analyses, as retinal band thickness pattern remains similar when individuals with low, medium or high physical activity were analysed separately (ESM Table 3).

It should be pointed out that we have carefully excluded any individual with pre-existing eye diseases and clinically relevant ophthalmological abnormalities prior to analyses. Furthermore, we have adjusted our models for relevant ophthalmological confounders further suggesting an independent link between glucose homeostasis and retinal layer characteristics in an approximately >50-fold larger cohort compared with previous publications. Thus, some, but not all, previous studies have found reduced global layer thicknesses in participants with type 2 diabetes for distinct bands in several small cohorts with or without manifest diabetic retinopathy compared to non-diabetic individuals [5, 9, 10, 37, 38]. Collectively, these data further support our results indicating glucose homeostasis is linked to lower retinal layer thicknesses.

A further strength of our study is the ETDRS-based analysis presenting location-specific pattern of the investigated markers. Here, ONL and MZ showed similar global and ETDRS subfield-specific associations, further validating these two layers as key metabolic areas of the retina.

It is interesting to note that in contrast to other retinal layers, IZ showed positive (with HOMA2-IR) and negative (with eGDR) associations with markers of insulin resistance/sensitivity in the ETDRS subfield-specific analysis. The IZ is the interacting area of photoreceptor outer segment tips and the apical microvilli border of the RPE cells, where phagocytosis of segment debris occurs [34] and where the RPE passes retinoids and other nutrients to the photoreceptors [39, 40]. Therefore, an increased IZ thickness could be representative of disturbed phagocytosis processes in disturbed glucose metabolism. Moreover, individuals with diabetes show stronger associations with retinal band thicknesses in the peri- and parafoveal subfields compared with the central foveal subfield A1, suggesting an eccentricity-dependence of glucose homeostasis on inner retinal effects [41].

This study has several limitations. Our study population predominantly consisted of European individuals, and, therefore, the findings may not be generalisable to populations of different ethnicities. Furthermore, owing to the cross-sectional design of this study at one baseline timepoint, no causal conclusions can be made. Moreover, our SD-OCT analyses do not include choroid, for which there is clear evidence of involvement in diabetic macular oedema and retinopathy [42, 43]. In contrast, to the best of our knowledge, this is the first study analysing static and dynamic continuous measures of glucose homeostasis, insulin resistance and sensitivity in a large number of deeply phenotyped individuals at a very high level of standardisation, as well as a thorough statistical approach accounting for several important covariates. Furthermore, we have incorporated principles of retinal neuroanatomy, vascular biology and OCT signal generation into our analyses to provide more mechanistical insights.

In conclusion, we demonstrate that glucose tolerance, insulin resistance and insulin sensitivity are associated with retinal layer thicknesses. The ONL and MZ show strongest associations with markers of glucose homeostasis, and changes in ONL and MZ thicknesses might predict early metabolic retinal alterations.

### Supplementary Information

Below is the link to the electronic supplementary material.

Supplementary file1 (PDF 1978 KB)

## Data Availability

Raw data cannot be shared publicly because of consent restrictions of LIFE-Adult-Study participants. Data are available after an approved project agreement from the LIFE Leipzig Research Center for Civilization Diseases. Please contact M. Nüchter (Head of Managing Office, contact via matthias.nuechter@life.uni-leipzig.de) for data access requests.

## Abbreviations

AUC_Glucose_: Area under the glucose curve
ΔBIC: Bayesian information criterion
CKD: Chronic kidney disease
eGDR: Estimated glucose disposal rate
ELM: External limiting membrane
ETDRS: Early Treatment Diabetic Retinopathy Study
EZ: Ellipsoid zone
EZ+OS: Ellipsoid zone + outer segment
GCL: Ganglion cell layer
HFL: Henle’s fibre layer
ILM: Internal limiting membrane
INL: Inner nuclear layer
IPL: Inner plexiform layer
IZ: Interdigitation zone
MCR: Metabolic clearance rate
MZ: Myoid zone
NGT: Normal glucose tolerance
OCT: Optical coherence tomography
ONL: Outer nuclear layer
OPL: Outer plexiform layer
OS: Outer segment
RNFL: Retinal nerve fibre layer
RPE: Retinal pigment epithelium
SD-OCT: Spectral domain optical coherence tomography
TyG index: Triglyceride glucose index
