# Supplementary material for: Glucose tolerance and insulin resistance/sensitivity associate with retinal layer characteristics: the LIFE-Adult-Study
Source: Diabetologia. 2024 Mar 2;67(5):928–39. doi: 10.1007/s00125-024-06093-9 (PMC10954961; doi:10.1007/s00125-024-06093-9)
Supplement: Supplementary file 1 — Supplementary file1 (PDF 1978 KB) [file 125_2024_6093_MOESM1_ESM.pdf]

ESM Methods

Data collection / Inclusion and Exclusion Criteria

B-scans with signal strength <20 dB were excluded from data analyses. The inclusion and exclusion process is depicted in ESM Figure 2. The aim of the present analysis was to investigate potential pathophysiological mechanisms prior to clinical onset of diabetes-induced eye diseases. Thus, we sequentially excluded subjects with missing SD-OCT scans (excluded N = 931), as well as eyes with any clinically significant retinal findings in the macular and optic nerve head regions (N = 1,616). For this purpose, two independent, experienced, and clinically trained observers analyzed OCT scans and fundus images. In case of inter-observer differences, a consensus decision was reached to classify the participant's eye. Clinical and sub-clinical ophthalmic findings were graded based on current ophthalmological standards. Eyes with clinical disease of the posterior eye within macula or optic nerve regions were excluded from the current study, i.e. all cases of retinal detachment or retinal hole, retinal pigment epithelium detachment, edema, bleeding, vascular abnormalities (such as vascular occlusion, ischemia, retinal vascular tortuosity, aneurysm, neovascularization), any kind of scarring, atrophy, fundus with disseminated white areas, cotton-wool-spots, fibrosis if traction or puckering with foveal involvement was observed. Subjects were also excluded if a tumor was present, or a staphyloma was detected. Within the macular region in specific, the following additional exclusion applied: age related macular degeneration (AMD) stages 2b, 3, 4a, and 4b, and maculopathy unrelated to AMD (stage 5), previously described by the Gutenberg Health Study [1] and based on the Rotterdam classification [2, 3]. Furthermore, subjects with missing diabetes diagnosis results (N = 69) were also excluded from data analyses, totaling to 2,616 excluded subjects (ESM Figure 1). For the remaining subjects (N = 7,384), one eye was randomly selected if both eyes of an included subject were reliable.

Anthropometric, biochemical markers, and markers of glucose homeostasis

At baseline, past medical history, as well as routine anthropometric (i.e. body mass index, blood pressure) measurements, were collected by trained study nurses. In a subcohort, a standardized 75g, oral glucose tolerance test was conducted with blood sampling at 0, 30, and 120 min [4]. In all subjects, fasting blood samples were drawn routinely. Total cholesterol, high density lipoprotein and low density lipoprotein cholesterol, triglycerides, glucose, insulin, glycated hemoglobin (HbA1c), cystatin C, and high-sensitivity c-reactive protein were quantified in a central lab by standard methods [4]. Estimated glomerular filtration rate was calculated using the cystatin C-based chronic kidney disease epidemiology collaboration equation [5]. Chronic kidney disease status was defined as a urinary albumin/creatinine ratio $\geqslant$ 30 mg/g and/or a decreased estimated glomerular filtration rate < 60 ml/min/1.73 m^2 [6].

Several markers of glucose homeostasis, insulin resistance, and insulin sensitivity were calculated based on oral glucose tolerance test-derived and fasting blood samples. Thus, area under the glucose curve $AUC_{glucose}$ was calculated according to the trapezoidal rule [7]. Homeostasis Model Assessment of beta cell function (HOMA2 %B) and insulin resistance (HOMA2 IR) were determined by HOMA2 Calculator software version 2.2.3 (University of Oxford, UK) using c-peptide levels. Only, if c-peptide was not available, insulin-based values were used. Triglyceride glucose index, estimated glucose disposal rate (eGDR), fasting Belfiore index, Stumvoll Insulin Sensitivity Index (0-30 min sampling and 0-120 min sampling), Stumvoll metabolic clearance rate (MCR, 0-120 min sampling), and McAuley index were calculated with standard equations depicted in ESM Table 1. Diabetes, prediabetes, as well as normal glucose tolerance (NGT), were defined according to the ADA definition [8]. Importantly, insulin sensitizing drugs potentially could influence the observed

results. However, only one single patient on thiazolidinediones treatment was identified in the current study cohort, thereby deeming thiazolidinediones as not relevant for our present analysis.

Physical activity level of each participant was determined by the International Physical Activity Questionnaire similar to Craig and co-workers [9], and categorized into low, moderate, or high physical activity.

Statistical Analysis

All statistical analyses were performed in R environment using version 4.0 (R Foundation for Statistical Computing, Vienna, Austria). For group-wise comparisons between subgroups of glucose homeostasis, as well as female vs. male subjects, ANOVA and/or unpaired student t-test (for continuous variables) or Chi-squared test (for categorical variables) were used, respectively.

We investigated the associations between a number of markers of glucose homeostasis and global average thickness of optical bands of the retina in the macula at baseline. For this purpose, multivariable linear regression analyses were carried out for the individual markers adjusted for age, sex, and refraction (i.e. scan focus) in all models. Prior to calculating the multivariate models, regression variables were checked for normal distribution. Using these covariates as independent variables in the respective models, the association of each marker of glucose homeostasis with the global thickness of each of the different optical macular bands (dependent variable) were calculated. Age, sex, and refraction were included as an independent variable in all models, since these covariates have been shown to confound the macular layer thickness measurements [10]. In case of missing data in any of the variables, no multiple imputation was performed and, therefore, no missing values for each regression variables were evident in each of the models.

Heatmaps of standardized β values from multivariable analyses for each global macular band thickness were produced for all glucose homeostasis parameters (Figure 1). To further identify whether a marker of glucose homeostasis is substantially related to the global thickness of a specific retinal bands, the increase of Bayesian information criterion (ΔBIC) > +2 was computed to compare a linear regression model containing age, sex, and refraction (model A) to an additional model comprising of model A + the respective marker of glucose homeostasis (model B) for each of the sectoral thickness of the optical bands. A ΔBIC > +2 was deemed as a relevant influence of the respective marker of glucose homeostasis to the linear regression model [11, 12].

We further associated ETDRS-based average subfield thickness for each of the optical macular bands with key markers of insulin resistance and sensitivity with subfield-specific linear regression models adjusted for age, sex, and refraction (Figure 2).

We carried out different sensitivity analyses stratifying our cohort by glucose homeostasis, i.e. normal glucose tolerance (NGT), prediabetes, and diabetes, according to the ADA definition [8] to provide more intuitive knowledge on clinical subgroups compared to overall linear modeling with interaction analyses. Furthermore, we have also re-analyzed our dataset using physical activity categories, neuroanatomical cell/tissue types, and ETDRS-based subfield categories, stratified by glucose homeostasis, respectively.

ESM Table 1: Overview on equations for the different markers of glucose homeostasis.

	Equation and units of measurement	Reference
**$AUC_{Glucose}$**	((Glucose 0min [mmol/l] + glucose 30min [mmol/l]) x 30 / 2) + ((glucose 30min + glucose 120min) x 90/2)	[7]
**TyG index**	ln((triglycerides [mmol/l] x 88.57) x (fasting glucose [pmol/l] x 18) / 2))	[13]
**eGDR**	21.158 + (-0.09 x waist circumference [cm]) + (-3.407 x presence of hypertension) + (-0.551 x HbA1c [%])	[14]
**Fasting Belfiore**	2 / ((fasting Insulin [pmol/l] x fasting glucose [mmol/l]) + 1)	[15]
**Stumvoll ISI (0-30)**	0.213 – 0.00305 × body mass index [kg/m²] – 0.000308 × fasting insulin [pmol/l] – 0.000640 × age [years]	[16]
**Stumvoll ISI (0-120)**	0.222 - 0.00333 × body mass index [kg/m²] – 0.0000799 × insulin 120min [pmol/l] – 0.000422 × age [years]	[16]
**Stumvoll MCR (0-120)**	19.240 – 0.281 × body mass index [kg/m²] – 0.00498 × insulin 120min [pmol/l] – 0.333 × glucose 120min [pmol/l]	[16]
**McAuley index**	exp[2.63 - 0.28 × ln(fasting insulin [pmol/l] / 6) - 0.31 × ln(tryglycerides [mmol/l])]	[17]

ESM Table 1: Overview on equations for the different markers of glucose homeostasis.

For each of the different markers of glucose homeostasis, the respective equation, as well as the measurement units and the reference, are depicted. $AUC_{Glucose}$, Area under the glucose curve; eGDR, estimated glucose disposal rate; ISI, Insulin sensitivity index; MCR, Metabolic clearance rate; TyG index, Triglyceride glucose index.

ESM Table 2: Retinal band thickness of all investigated bands stratified by sex and glucose homeostasis (N=7,384)

	Entire cohort		p between sexes	Normal glucose tolerance		Prediabetes		Diabetes		p between 3 glucose subgroups	
**Total N**	3,933	3,451	-	2,559	1,650	951	1,275	423	526	-	-
**Sex** / **Band**	♀	♂	**-**	♀	♂	♀	♂	♀	♂	♀	♂
**RNFL (µm)**	39.2 ± 5.2	37.9 ± 4.9	**<0.001**	39.5 ± 5.2	38.3 ± 4.7	38.9 ± 5.1	37.7 ± 5.0	38.1 ± 4.8	37.0 ± 5.1	**<0.001**	**<0.001**
**GCL (µm)**	34.8 ± 3.1	35.1 ± 3.3	**0.004**	35.1 ± 3.0	35.5 ± 3.2	34.6 ± 3.2	34.9 ± 3.4	33.9 ± 3.2	34.0 ± 3.4	**<0.001**	**<0.001**
**IPL (µm)**	28.5 ± 2.3	28.7 ± 2.5	**0.003**	28.7 ± 2.3	28.9 ± 2.4	28.4 ± 2.4	28.6 ± 2.5	28.0 ± 2.3	28.0 ± 2.6	**<0.001**	**<0.001**
**INL (µm)**	31.9 ± 2.1	32.5 ± 2.3	**<0.001**	32.1 ± 2.1	32.6 ± 2.3	31.8 ± 2.2	32.5 ± 2.3	31.6 ± 2.1	32.1 ± 2.2	**<0.001**	**<0.001**
**OPL (µm)**	27.9 ± 2.0	28.0 ± 2.0	0.074	27.9 ± 2.0	27.9 ± 2.1	27.9 ± 2.0	28.0 ± 1.9	27.9 ± 1.9	28.0 ± 2.1	0.702	0.500
**ONL (µm)**	58.9 ± 6.3	60.1 ± 6.4	**<0.001**	59.3 ± 6.2	60.6 ± 6.3	58.4 ± 6.4	59.9 ± 6.4	57.5 ± 6.5	58.6 ± 6.6	**<0.001**	**<0.001**
**MZ (µm)**	23.7 ± 0.7	23.6 ± 0.5	**<0.001**	23.8 ± 0.7	23.7 ± 0.6	23.6 ± 0.5	23.6 ± 0.5	23.5 ± 0.4	23.5 ± 0.3	**<0.001**	**<0.001**
**EZ+OS (µm)**	18.2 ± 1.5	18.9 ± 1.8	**<0.001**	18.3 ± 1.5	19.1 ± 1.7	17.9 ± 1.4	18.9 ± 1.8	17.7 ± 1.4	18.5 ± 1.9	**<0.001**	**<0.001**
**IZ (µm)**	22.4 ± 1.4	22.5 ± 1.5	**<0.001**	22.2 ± 1.4	22.4 ± 1.4	22.5 ± 1.4	22.6 ± 1.5	22.7 ± 1.4	22.8 ± 1.6	**<0.001**	**<0.001**
**RPE (µm)**	14.0 ± 1.5	14.2 ± 1.1	**<0.001**	14.0 ± 1.6	14.2 ± 1.1	14.0 ± 1.2	14.1 ± 1.1	13.9 ± 1.5	14.1 ± 1.1	0.315	**0.002**

ESM Table 2: Retinal band thicknesses stratified by sex and glucose homeostasis (N = 7,384).

Retinal nerve fibre layer (RNFL); Ganglion cell layer (GCL); Inner plexiform layer (IPL); Inner nuclear layer (INL); Outer plexiform layer (OPL); Outer nuclear layer (ONL) (including thickness of external limiting membrane (ELM), see Methods section for further details); Myoid zone (MZ); Ellipsoid zone (EZ) and outer-photoreceptor segment (OS) combined (named EZ+OS here) (see Methods section for further details); Interdigitation zone (IZ); Retinal pigment epithelium (RPE). Values for mean ± standard deviation are shown. Differences in retinal band thicknesses between both sexes in the entire cohort were investigated using t tests and exact p-values are given. Overall sex-stratified

differences in retinal band thicknesses between the three groups of glucose homeostasis, i.e. normal glucose tolerance vs. prediabetes vs. diabetes, were assessed by ANOVA. Sex-specific p-values after correction for multiple testing based on the false discovery rate method are depicted. Bold numbers indicate $p<0.05$ for all investigated comparisons.

ESM Table 3: Retinal band thickness of all investigated layers stratified by physical activity categories by International Physical Activity Questionnaire (IPAQ) and glucose homeostasis (N=7,384)

	**Entire cohort**				**Normal glucose tolerance**			**Prediabetes**			**Diabetes**			**p between 3 glucose subgroups**		
**Physical activity**	Low	Mod-erate	High	**p**	Low	Mod-erate	High	Low	Mod-erate	High	Low	Mod-erate	High	Low	Mod-erate	High
**RNFL (µm)**	38.5 ± 5.1	38.9 ± 5.0	38.7 ± 5.1	0.920	38.8 ± 5.0	39.5 ± 5.0	39.0 ± 5.1	38.3 ± 5.2	38.0 ± 4.9	38.4 ± 5.1	37.3 ± 5.5	37.9 ± 5.0	37.5 ± 4.9	**0.005**	**<0.001**	**<0.001**
**GCL (µm)**	34.9 ± 3.1	35.0 ± 3.2	35.1 ± 3.1	0.146	35.1 ± 2.9	35.2 ± 3.2	35.4 ± 3.0	35.0 ± 3.4	34.8 ± 3.3	34.8 ± 3.2	33.9 ± 3.2	34.2 ± 3.1	34.0 ± 3.3	**0.002**	**<0.001**	**<0.001**
**IPL (µm)**	28.5 ± 2.4	28.6 ± 2.4	28.7 ± 2.3	**0.046**	28.6 ± 2.3	28.7 ± 2.4	28.8 ± 2.3	28.6 ± 2.5	28.6 ± 2.4	28.5 ± 2.4	27.8 ± 2.4	28.2 ± 2.3	28.1 ± 2.5	**0.006**	**0.005**	**<0.001**
**INL (µm)**	32.1 ± 2.2	32.2 ± 2.2	32.3 ± 2.2	**0.049**	32.1 ± 2.3	32.2 ± 2.2	32.3 ± 2.2	32.2 ± 2.2	32.2 ± 2.2	32.2 ± 2.3	31.7 ± 1.9	32.1 ± 2.1	31.9 ± 2.1	0.110	0.641	**<0.001**
**OPL (µm)**	27.7 ± 1.9	27.9 ± 2.0	27.9 ± 2.0	**0.001**	27.6 ± 2.0	27.9 ± 2.1	27.9 ± 2.0	27.7 ± 1.9	27.9 ± 2.0	28.0 ± 2.0	27.9 ± 2.0	27.9 ± 2.1	28.0 ± 2.0	0.168	0.824	0.115
**ONL (µm)**	59.5 ± 6.0	59.5 ± 6.3	59.6 ± 6.4	0.875	59.9 ± 6.0	59.7 ± 6.3	60.0 ± 6.3	59.4 ± 6.1	59.5 ± 6.4	59.2 ± 6.4	58.2 ± 5.9	58.7 ± 6.4	58.1 ± 6.7	**0.009**	0.074	**<0.001**
**MZ (µm)**	23.7 ± 0.6	23.7 ± 0.6	23.7 ± 0.6	0.096	23.8 ± 0.7	23.8 ± 0.7	23.8 ± 0.7	23.5 ± 0.4	23.6 ± 0.5	23.6 ± 0.5	23.5 ± 0.3	23.5 ± 0.3	23.5 ± 0.3	**<0.001**	**<0.001**	**<0.001**
**EZ+OS (µm)**	18.6 ± 1.7	18.5 ± 1.7	18.6 ± 1.7	0.072	18.7 ± 1.6	18.6 ± 1.6	18.7 ± 1.6	18.5 ± 1.7	18.5 ± 1.7	18.5 ± 1.8	18.2 ± 1.7	18.0 ± 1.6	18.3 ± 1.8	**0.010**	**<0.001**	**<0.001**
**IZ (µm)**	22.4 ± 1.5	22.5 ± 1.4	22.4 ± 1.4	0.376	22.2 ± 1.5	22.3 ± 1.4	22.2 ± 1.4	22.6 ± 1.5	22.6 ± 1.5	22.5 ± 1.5	22.7 ± 1.5	22.7 ± 1.5	22.8 ± 1.5	**<0.001**	**<0.001**	**<0.001**
**RPE (µm)**	14.0 ± 1.0	14.0 ± 1.1	14.1 ± 1.3	**0.036**	14.0 ± 1.0	14.0 ± 1.1	14.1 ± 1.3	13.9 ± 1.0	14.1 ± 1.2	14.1 ± 1.1	13.9 ± 1.1	14.0 ± 1.1	14.0 ± 1.5	0.240	0.557	0.641

ESM Table 3: Retinal band thickness of all investigated layers stratified by physical activity categories by the International Physical Activity Questionnaire (IPAQ) and glucose homeostasis.

IPAQ-derived physical activity categories were defined similar to Craig and co-workers [9] and stratified into low, moderate, or high. Abbreviations as indicated in ESM Table 2. Values for mean ± standard deviation are shown. Differences in retinal band thicknesses between the three physical activity categories in the entire cohort were investigated using ANOVA and exact p-values are given. Specifically for individuals with low, moderate, or high physical activity, respectively, overall differences in retinal band thicknesses between the three groups of glucose homeostasis (i.e. normal glucose tolerance vs. prediabetes vs. diabetes) were also assessed by ANOVA. Physical activity category-specific p-values after correction for multiple testing based on the false discovery rate method are depicted. Bold numbers indicate p<0.05 for all investigated comparisons.

ESM Table 4: Grouped retinal band thicknesses representing relevant retinal cell classes of ganglion cells, the intrinsic retinal vasculature, as well as photoreceptor stratified by sex and glucose homeostasis

	Entire cohort		p between sexes	Normal glucose tolerance		Prediabetes		Diabetes		p between 3 glucose subgroups	
**Sex** / **Cell / tissue classes**	♀	♂	-	♀	♂	♀	♂	♀	♂	♀	♂
**Ganglion cell-containing bands (µm)**	34.2 ± 2.7	33.9 ± 2.9	**<0.001**	34.4 ± 2.7	34.2 ± 2.8	34.0 ± 2.7	33.7 ± 3.0	33.3 ± 2.8	33.0 ± 3.1	**<0.001**	**<0.001**
**Bands containing the intrinsic retinal vasculature (µm)**	32.5 ± 2.0	32.4 ± 2.1	0.186	32.6 ± 2.0	32.7 ± 2.1	32.3 ± 2.0	32.3 ± 2.2	31.9 ± 2.0	31.8 ± 2.2	**<0.001**	**<0.001**
**Photoreceptor-containing bands (µm)**	30.2 ± 1.4	30.6 ± 1.4	**<0.001**	30.3 ± 1.4	30.8 ± 1.4	30.1 ± 1.4	30.6 ± 1.4	29.9 ± 1.4	30.3 ± 1.5	**<0.001**	**<0.001**

ESM Table 4: Grouped retinal band thicknesses representing relevant retinal cell classes of ganglion cells, the intrinsic retinal vasculature, as well as photoreceptor stratified by sex and glucose homeostasis.

Retinal band thicknesses of different layers were grouped for all ganglion cell-containing layers (i.e. RNFL, GCL, and IPL), all layers containing the intrinsic retinal vasculature (i.e. RNFL, GCL, IPL, INL, and OPL), as well as all photoreceptor-containing bands (i.e. OPL, ONL, MZ, EZ+OS, and IZ). Abbreviations as indicated in ESM Table 2. Values for mean ± standard deviation are shown. Differences in the thicknesses of these grouped retinal band for ganglion cells, the intrinsic retinal vasculature, and photoreceptors between women and men in the entire cohort were investigated using Student's t test and exact p-values are given. Specifically for female and male individuals, overall differences in retinal band thicknesses between the three groups of glucose homeostasis (i.e. normal glucose tolerance vs. prediabetes vs. diabetes) were also assessed by ANOVA. Sex-specific p-values after correction for multiple testing based on the false discovery rate method are depicted. Bold numbers indicate $p<0.05$ for all investigated comparisons.

ESM Table 5: Retinal band thickness of all investigated bands stratified by type of diabetes

Total N	22	915	
**Diabetes Type**<br>**Band**	Type 1 diabetes	Type 2 diabetes	**p**
**RNFL (µm)**	36.2 ± 6.5	37.5 ± 4.9	0.907
**GCL (µm)**	33.8 ± 4.4	34.0 ± 3.3	0.970
**IPL (µm)**	28.0 ± 2.5	28.0 ± 2.5	0.970
**INL (µm)**	31.9 ± 1.8	31.8 ± 2.2	0.970
**OPL (µm)**	28.0 ± 1.6	28.0 ± 2.0	0.970
**ONL (µm)**	60.7 ± 7.7	58.1 ± 6.5	0.610
**MZ (µm)**	23.6 ± 0.4	23.5 ± 0.3	0.907
**EZ+OS (µm)**	18.3 ± 1.9	18.1 ± 1.7	0.970
**IZ (µm)**	22.8 ± 1.7	22.8 ± 1.5	0.970
**RPE (µm)**	14.6 ± 1.2	14.0 ± 1.3	0.275

ESM Table 5: Retinal band thickness of all investigated bands stratified by type of diabetes.

Retinal band thicknesses of different layers were compared between individuals with type 1 diabetes and type 2 diabetes. Abbreviations as indicated in ESM Table 2. Values for mean ± standard deviation are shown. Differences in the thicknesses of the retinal bands between both types of diabetes were investigated using Student’s t test and exact p-values are given.

ESM Table 6: Retinal band thickness of all investigated layers stratified by ETDRS subfield categories and glucose homeostasis (N=7,384)

	**Entire cohort**				**Normal glucose tolerance**			**Prediabetes**			**Diabetes**			**p between 3 glucose subgroups**		
**Retinal subfields**<br>**Bands**	**Fovea**	**Para-foveal**	**Peri-foveal**	**p**	**Fovea**	**Para-foveal**	**Peri-foveal**	**Fovea**	**Para-foveal**	**Peri-foveal**	**Fovea**	**Para-foveal**	**Peri-foveal**	**Fovea subfield only**	**Para-foveal subfields only**	**Peri-foveal subfields only**
**RNFL (µm)**	13.5 ± 8.7	23.5 ± 3.4	42.6 ± 5.9	**<0.001**	13.4 ± 10.1	23.6 ± 3.4	43.1 ± 5.8	13.6 ± 7.6	23.4 ± 3.7	42.1 ± 5.8	13.5 ± 2.9	23.1 ± 2.4	41.3 ± 5.7	0.737	**<0.001**	**<0.001**
**GCL (µm)**	16.9 ± 6.2	50.2 ± 5.3	32.1 ± 3.1	**<0.001**	16.8 ± 6.0	50.8 ± 5.0	32.4 ± 3.0	16.9 ± 6.3	49.8 ± 5.4	32.0 ± 3.2	16.9 ± 6.8	48.2 ± 5.6	31.4 ± 3.1	0.712	**<0.001**	**<0.001**
**IPL (µm)**	22.6 ± 4.4	41.1 ± 3.5	26.0 ± 2.3	**<0.001**	22.6 ± 4.3	41.5 ± 3.4	26.2 ± 2.3	22.7 ± 4.4	41.0 ± 3.6	26.0 ± 2.4	22.5 ± 4.7	39.9 ± 3.7	25.6 ± 2.3	0.618	**<0.001**	**<0.001**
**INL (µm)**	21.0 ± 6.3	40.4 ± 3.4	30.7 ± 2.2	**<0.001**	20.5 ± 6.0	40.3 ± 3.3	30.9 ± 2.2	21.6 ± 6.3	40.6 ± 3.5	30.7 ± 2.2	22.2 ± 6.9	40.3 ± 3.4	30.3 ± 2.1	**<0.001**	0.497	**<0.001**
**OPL (µm)**	26.3 ± 5.8	33.1 ± 4.1	26.9 ± 1.8	**<0.001**	26.2 ± 5.7	33.1 ± 4.1	26.8 ± 1.8	26.3 ± 5.6	33.1 ± 4.0	26.9 ± 1.8	26.6 ± 6.3	33.2 ± 4.2	26.9 ± 1.8	0.054	0.630	0.061
**ONL (µm)**	93.5 ± 10.6	71.6 ± 8.1	55.8 ± 6.2	**<0.001**	93.3 ± 10.2	71.8 ± 8.0	56.3 ± 6.1	94.0 ± 10.8	71.8 ± 8.1	55.6 ± 6.3	92.8 ± 11.5	70.7 ± 8.5	54.5 ± 6.3	0.915	**0.006**	**<0.001**
**MZ (µm)**	27.3 ± 3.0	24.3 ± 1.2	23.4 ± 0.5	**<0.001**	27.5 ± 3.0	24.5 ± 1.3	23.5 ± 0.6	27.1 ± 2.9	24.2 ± 1.1	23.4 ± 0.4	26.5 ± 2.8	23.9 ± 0.8	23.3 ± 0.2	**<0.001**	**<0.001**	**<0.001**
**EZ+OS (µm)**	21.9 ± 4.0	19.2 ± 2.2	18.3 ± 1.6	**<0.001**	22.2 ± 3.9	19.4 ± 2.1	18.4 ± 1.6	21.8 ± 4.2	19.1 ± 2.3	18.2 ± 1.7	20.9 ± 4.1	18.6 ± 2.3	18.0 ± 1.6	**<0.001**	**<0.001**	**<0.001**
**IZ (µm)**	22.7 ± 1.8	22.1 ± 1.8	22.5 ± 1.4	**<0.001**	22.5 ± 1.8	21.9 ± 1.7	22.4 ± 1.4	22.9 ± 1.9	22.2 ± 1.8	22.6 ± 1.4	23.1 ± 1.9	22.5 ± 1.8	22.8 ± 1.5	**<0.001**	**<0.001**	**<0.001**
**RPE (µm)**	17.3 ± 2.2	15.6 ± 1.6	13.6 ± 1.3	**<0.001**	17.5 ± 2.1	15.6 ± 1.5	13.6 ± 1.5	17.2 ± 2.4	15.6 ± 1.5	13.6 ± 1.1	17.0 ± 2.4	15.5 ± 2.2	13.6 ± 1.2	**<0.001**	0.058	0.275

ESM Table 6: Retinal band thickness of all investigated layers stratified by ETDRS subfield categories and glucose homeostasis.

Retinal subfield categories were defined as follows: Foveal subfield: A1; Parafoveal subfields: A2+A3+A4+A5 from the inner ring of the ETDRS grid; Perifoveal subfields: A6+A7+A8+A9 from the outer ring of the ETDRS grid. Abbreviations as indicated in ESM Table 2. Values for mean ± standard deviation are shown. Differences in retinal band thicknesses between the three subfield categories in the entire cohort were investigated using ANOVA and exact p-values are given. Fovea-only, parafoveal-only, and perifoveal-only overall differences in retinal band thicknesses between the three groups of glucose homeostasis (i.e. normal glucose tolerance vs. prediabetes vs. diabetes) were also assessed by ANOVA. ETDRS subfield category-specific p-values after correction for multiple testing based on the false discovery rate method are depicted. Bold numbers indicate p<0.05 for all investigated comparisons.

ESM Fig. 1

Normal glucose tolerance

A B

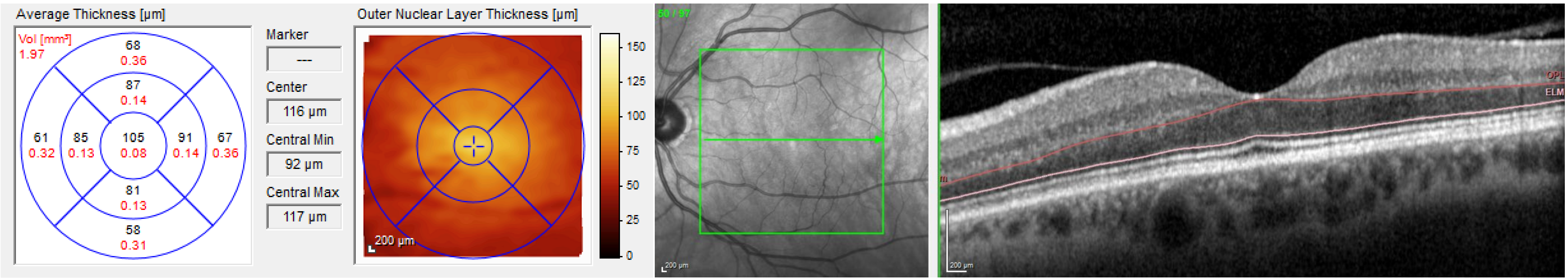

Diabetes

A B

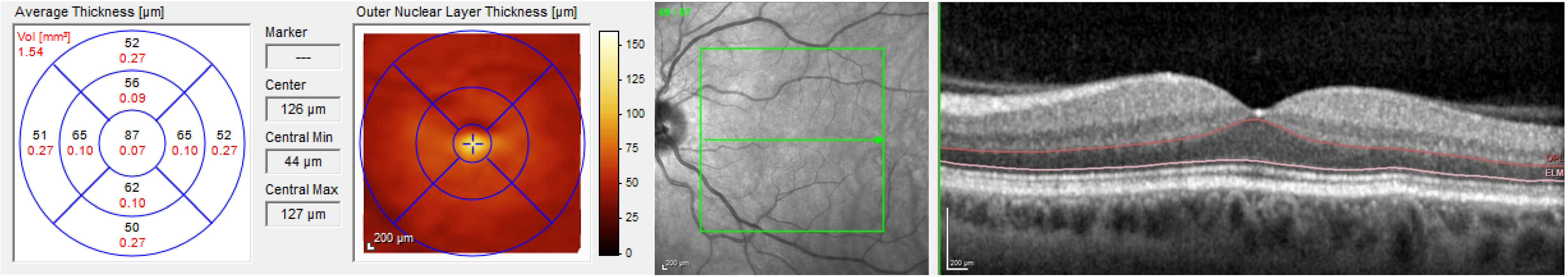

ESM Figure 1

Representative SD-OCT data (male, equal refractive error, equal age) from an individual with normal glucose tolerance (NGT; upper panel) and from a participant with diabetes (lower panel). **A)** Early Treatment Diabetic Retinopathy Study (ETDRS) subfields-specific volume and layer thicknesses of the Outer nuclear layer (ONL), i.e. the area below the Outer plexiform layer (OPL) and including the thickness of external limiting membrane (ELM; see Methods section for further details). Furthermore, a heatmap of subfield-specific ONL thickness is depicted. **B)** Representative B-scan images corresponding to the same individuals are depicted, respectively. Image acquisition and segmentation was performed by HEYEX software (Heidelberg Engineering, Heidelberg, Germany).

ESM Fig. 2

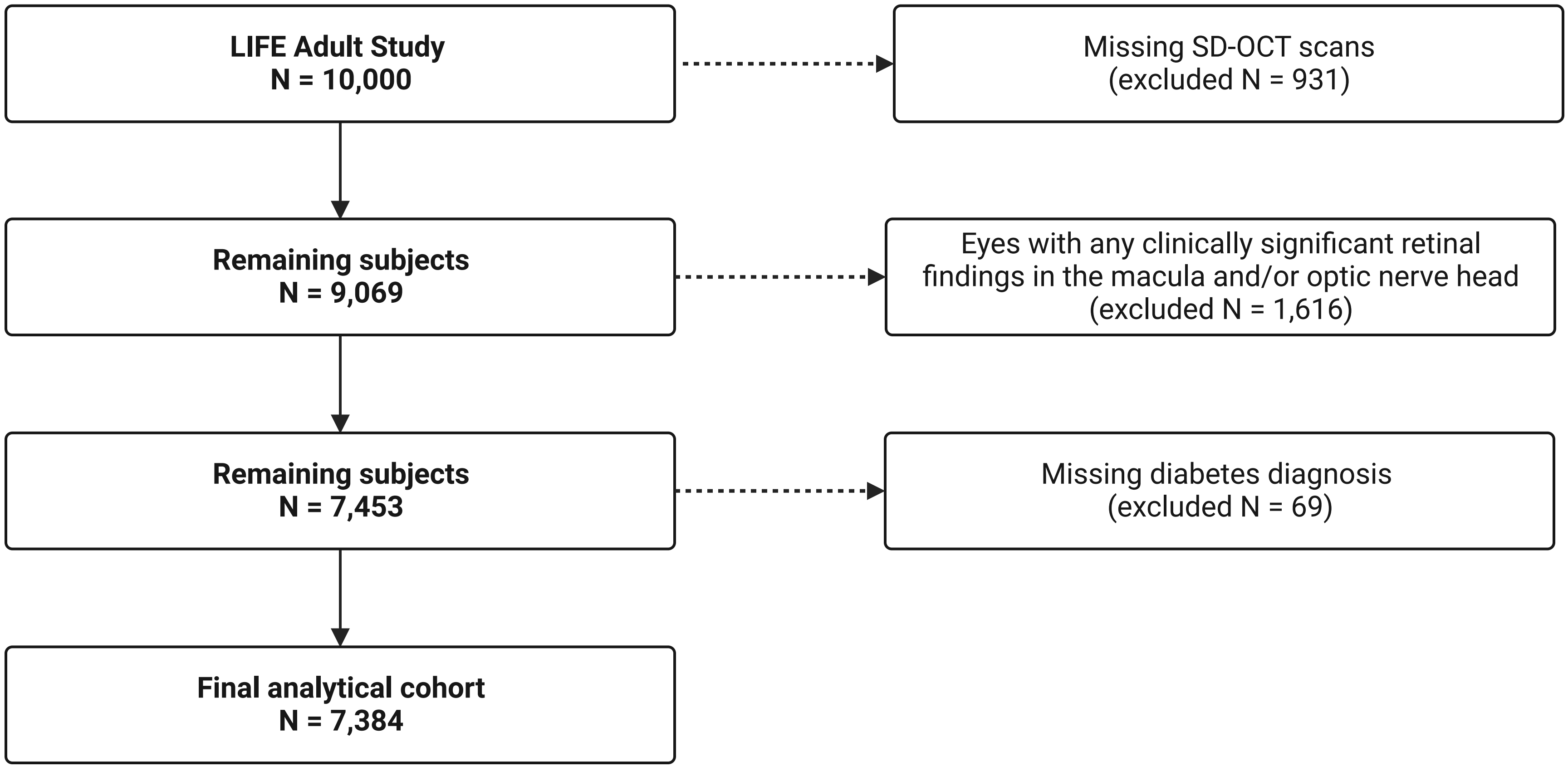
LIFE Adult Study
N = 10,000
Missing SD-OCT scans
(excluded N = 931)
Remaining subjects
N = 9,069
Eyes with any clinically significant retinal findings in the macula and/or optic nerve head
(excluded N = 1,616)
Remaining subjects
N = 7,453
Missing diabetes diagnosis
(excluded N = 69)
Final analytical cohort
N = 7,384

ESM Figure 2

Flowchart illustrating the subject exclusion process. Of the 10,000 participants initially recruited in the LIFE-Adult-Study, SD-OCT scans were missing for 931 subjects. Of the remaining 9,069 subjects, 1,616 individuals were excluded due to any clinically relevant retinal findings in the macular and optic nerve head regions. Sequentially, 69 subjects with missing diabetes diagnosis results were excluded from further analyses. After the exclusion process, 7,384 eyes were available for data analysis.

LIFE, The Leipzig Research Centre for Civilization Diseases; SD-OCT, Spectral domain optical coherence tomography.

ESM Fig. 3

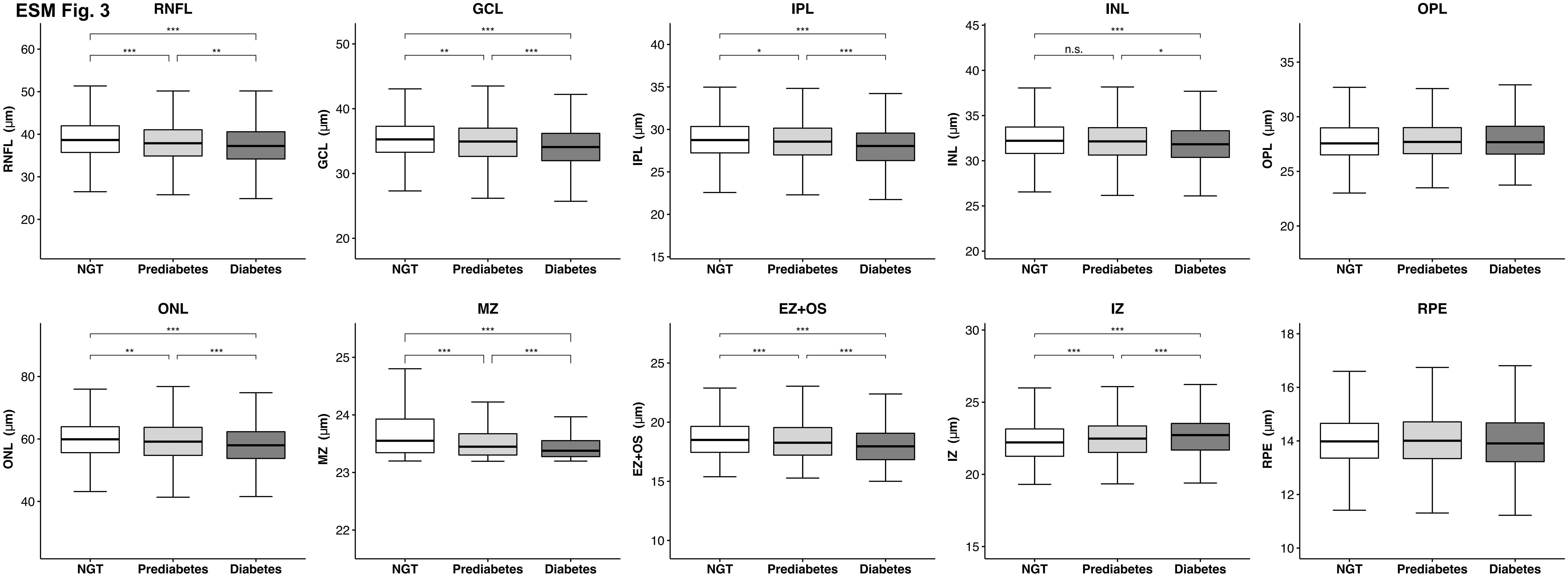
RNFL
GCL
IPL
INL
OPL
ONL
MZ
EZ+OS
IZ
RPE
RNFL (µm)
GCL (µm)
IPL (µm)
INL (µm)
OPL (µm)
ONL (µm)
MZ (µm)
EZ+OS (µm)
IZ (µm)
RPE (µm)
NGT
Prediabetes
Diabetes
n.s.

ESM Figure 3

Boxplots of retinal layer thicknesses of all investigated bands stratified by glucose homeostasis. Differences in retinal layer thicknesses between the three subgroups of glucose homeostasis were investigated using ANOVA with post-hoc tests for group-wise comparisons, and p-values after correction for multiple testing based on the false discovery rate method are depicted as follows: *** denotes $p < 0.001$; ** denotes $p < 0.01$; * denotes $p < 0.05$; n.s., not significant. Abbreviations: EZ, Ellipsoid zone and outer-photoreceptor segment (OS) combined (named EZ+OS here) (see Methods section for further details); GCL, Ganglion cell layer; INL, Inner nuclear layer; IPL, Inner plexiform layer; IZ, Interdigitation zone; MZ, Myoid zone; NGT, Normal glucose tolerance; ONL, Outer nuclear layer (including thickness of external limiting membrane (ELM; see Methods section for further details); OPL, Outer plexiform layer; RNFL, Retinal nerve fiber layer; RPE, Retinal pigment epithelium.

ESM Fig. 4

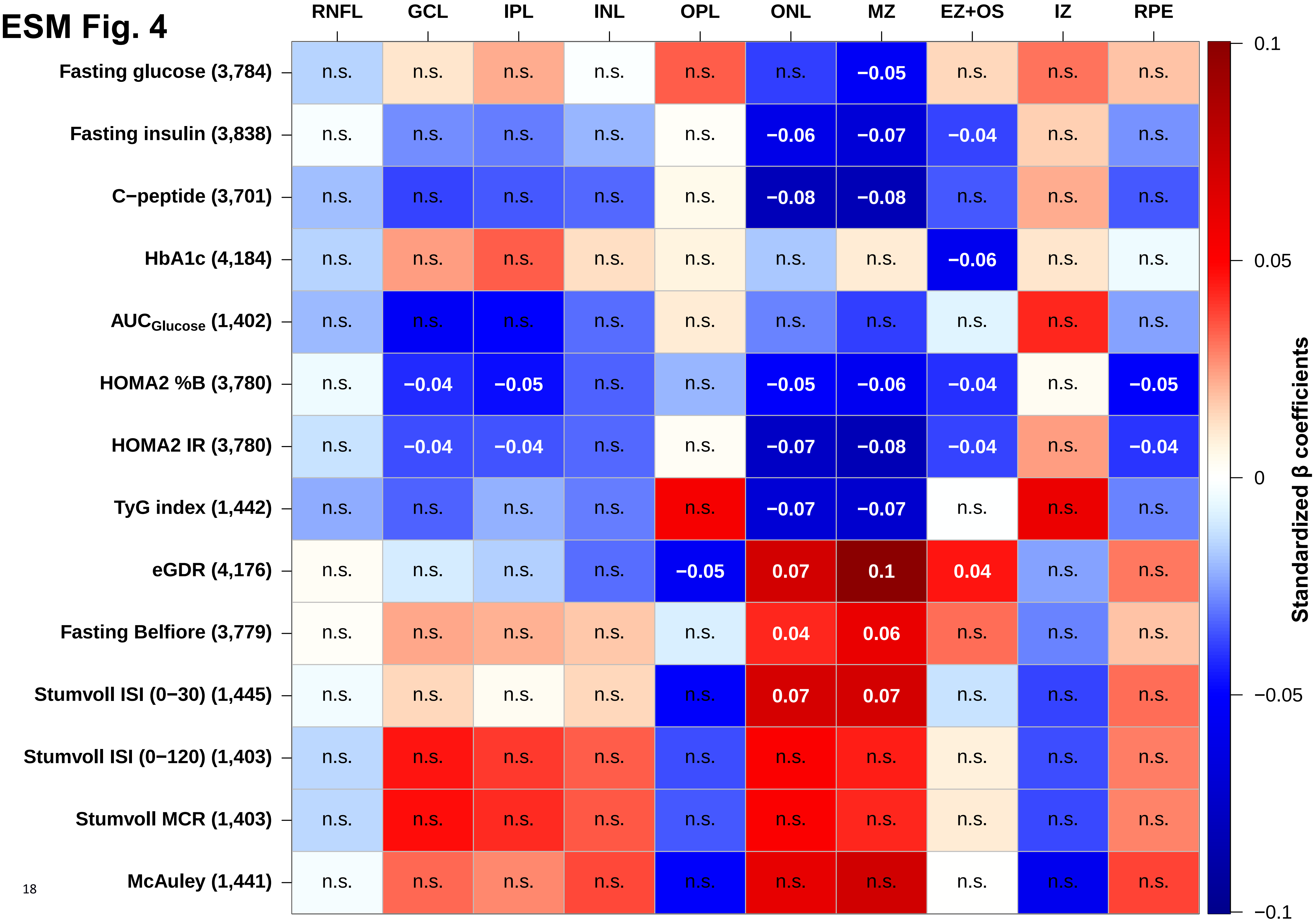

RNFL GCL IPL INL OPL ONL MZ EZ+OS IZ RPE
Fasting glucose (3,784): n.s. n.s. n.s. n.s. n.s. n.s. −0.05 n.s. n.s. n.s.
Fasting insulin (3,838): n.s. n.s. n.s. n.s. n.s. −0.06 −0.07 −0.04 n.s. n.s.
C−peptide (3,701): n.s. n.s. n.s. n.s. n.s. −0.08 −0.08 n.s. n.s. n.s.
HbA1c (4,184): n.s. n.s. n.s. n.s. n.s. n.s. n.s. −0.06 n.s. n.s.
AUC$_{Glucose}$ (1,402): n.s. n.s. n.s. n.s. n.s. n.s. n.s. n.s. n.s. n.s.
HOMA2 %B (3,780): n.s. −0.04 −0.05 n.s. n.s. −0.05 −0.06 −0.04 n.s. −0.05
HOMA2 IR (3,780): n.s. −0.04 −0.04 n.s. n.s. −0.07 −0.08 −0.04 n.s. −0.04
TyG index (1,442): n.s. n.s. n.s. n.s. n.s. −0.07 −0.07 n.s. n.s. n.s.
eGDR (4,176): n.s. n.s. n.s. n.s. −0.05 0.07 0.1 0.04 n.s. n.s.
Fasting Belfiore (3,779): n.s. n.s. n.s. n.s. n.s. 0.04 0.06 n.s. n.s. n.s.
Stumvoll ISI (0−30) (1,445): n.s. n.s. n.s. n.s. n.s. 0.07 0.07 n.s. n.s. n.s.
Stumvoll ISI (0−120) (1,403): n.s. n.s. n.s. n.s. n.s. n.s. n.s. n.s. n.s. n.s.
Stumvoll MCR (1,403): n.s. n.s. n.s. n.s. n.s. n.s. n.s. n.s. n.s. n.s.
McAuley (1,441): n.s. n.s. n.s. n.s. n.s. n.s. n.s. n.s. n.s. n.s.
0.1
0.05
0
−0.05
−0.1
Standardized β coefficients

ESM Figure 4

Heatmap of standardized β coefficients for all investigated markers of glucose homeostasis and the 10 different retinal layer thicknesses **in individuals with normal glucose tolerance only (N=4,209)**. Separate multivariable linear regression analyses were carried out for each of the markers (independent variable) and the respective retinal layer thickness (dependent variable). All multivariable models were adjusted for age, sex, and refraction. The false-positive discovery rate method was applied to correct p-values for multiple comparisons. For all multivariable models, strength as assessed by standardized β, as well as the direction, of the associations are color-coded. Thus, positive (in red/warmer colors) and negative (in blue/cooler colors) associations are shaded based on the respective standardized β coefficients. The exact standardized β coefficients is given for all significant models with $p<0.05$. If the respective linear regression model did not show an overall significance (indicating that the standardized β is not valid for this association), no exact standardized β coefficients are depicted. Number of subjects included in all multivariate models for the respective marker of glucose homeostasis are provided.

$AUC_{Glucose}$, Area under the glucose curve; eGDR, estimated glucose disposal rate; HbA1c, Glycated hemoglobin A1c; HOMA2 %B, Homeostasis Model Assessment of beta cell function; HOMA2 IR Homeostasis Model Assessment of Insulin resistance; TyG index, Triglyceride glucose index. All other abbreviations are indicated in ESM Figure 3.

ESM Fig. 5

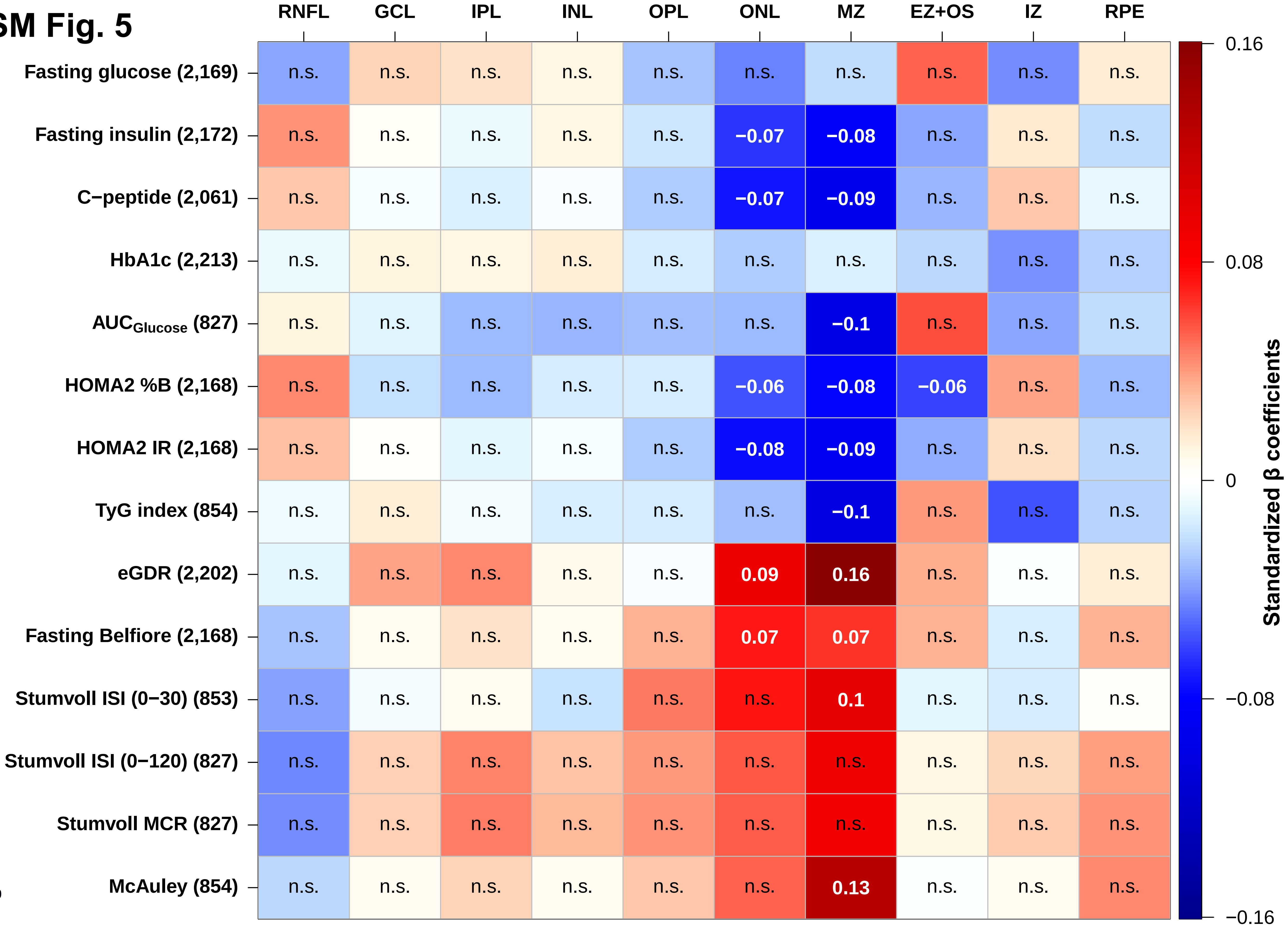

RNFL
GCL
IPL
INL
OPL
ONL
MZ
EZ+OS
IZ
RPE
Fasting glucose (2,169)
Fasting insulin (2,172)
C−peptide (2,061)
HbA1c (2,213)
AUC$_{Glucose}$ (827)
HOMA2 %B (2,168)
HOMA2 IR (2,168)
TyG index (854)
eGDR (2,202)
Fasting Belfiore (2,168)
Stumvoll ISI (0−30) (853)
Stumvoll ISI (0−120) (827)
Stumvoll MCR (827)
McAuley (854)
n.s.
−0.07
−0.08
−0.07
−0.09
−0.1
−0.06
−0.08
−0.06
−0.08
−0.09
−0.1
0.09
0.16
0.07
0.07
0.1
0.13
Standardized β coefficients
0.16
0.08
0
−0.08
−0.16

ESM Figure 5

Heatmap of standardized β coefficients for all investigated markers of glucose homeostasis and the 10 different retinal layer thicknesses in **individuals with prediabetes only (N=2,226)**. Separate multivariable linear regression analyses were carried out for each of the markers (independent variable) and the respective retinal layer thickness (dependent variable). All multivariable models were adjusted for age, sex, and refraction. The false-positive discovery rate method was applied to correct p-values for multiple comparisons. For all multivariable models, strength as assessed by standardized β, as well as the direction, of the associations are color-coded. Thus, positive (in red/warmer colors) and negative (in blue/cooler colors) associations are shaded based on the respective standardized β coefficients. The exact standardized β coefficients is given for all significant models with $p<0.05$. If the respective linear regression model did not show an overall significance (indicating that the standardized β is not valid for this association), no exact standardized β coefficients are depicted. Number of subjects included in all multivariate models for the respective marker of glucose homeostasis are provided.

Abbreviations are indicated in ESM Figure 3.

ESM Fig. 6

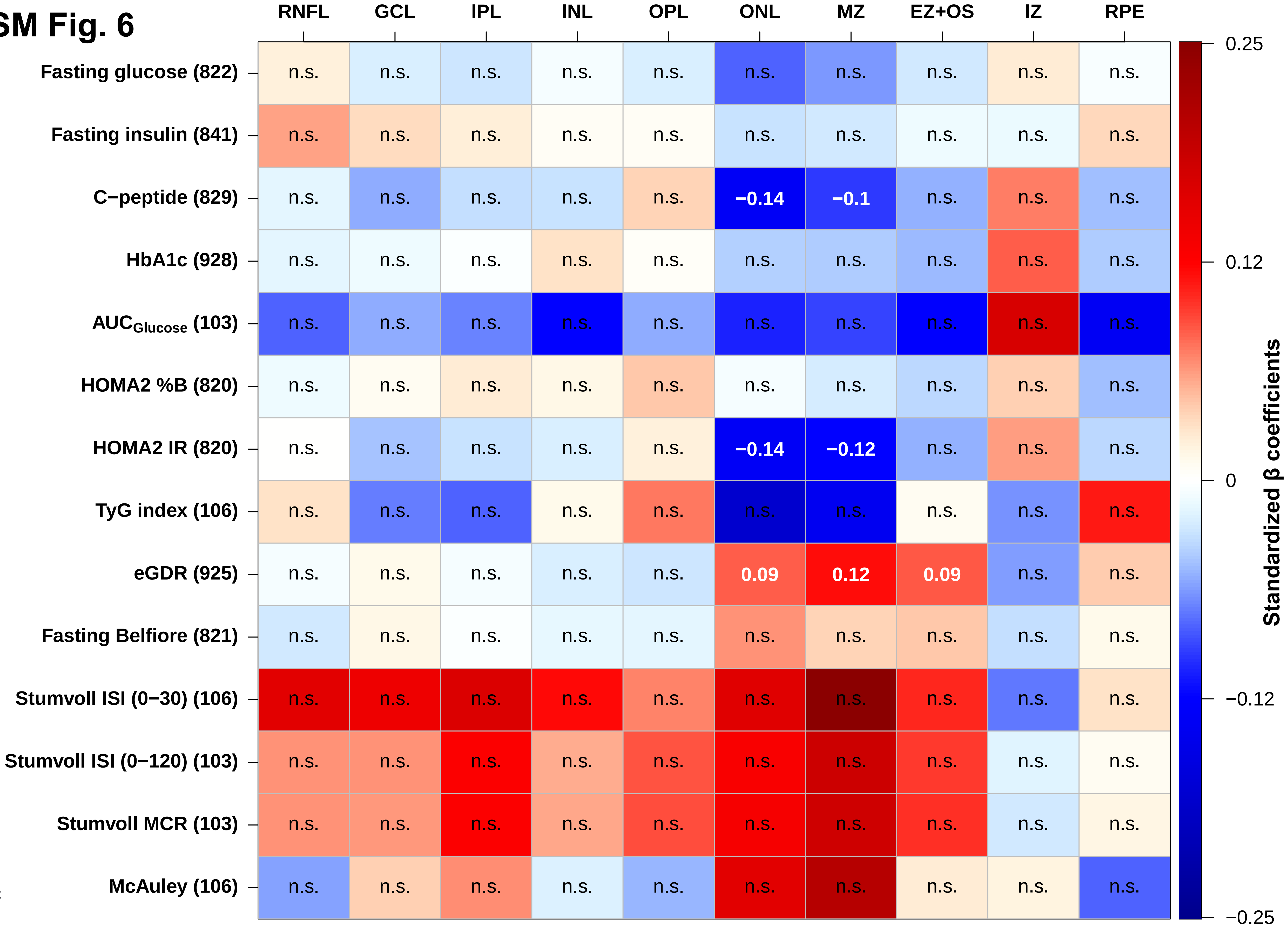

RNFL
GCL
IPL
INL
OPL
ONL
MZ
EZ+OS
IZ
RPE
Fasting glucose (822)
Fasting insulin (841)
C−peptide (829)
HbA1c (928)
AUCGlucose (103)
HOMA2 %B (820)
HOMA2 IR (820)
TyG index (106)
eGDR (925)
Fasting Belfiore (821)
Stumvoll ISI (0−30) (106)
Stumvoll ISI (0−120) (103)
Stumvoll MCR (103)
McAuley (106)
n.s.
−0.14
−0.1
−0.14
−0.12
0.09
0.12
0.09
0.25
0.12
0
−0.12
−0.25
Standardized β coefficients

ESM Figure 6

Heatmap of standardized β coefficients for all investigated markers of glucose homeostasis and the 10 different retinal layer thicknesses in **individuals with diabetes only (N=949)**. Separate multivariable linear regression analyses were carried out for each of the markers (independent variable) and the respective retinal layer thickness (dependent variable). All multivariable models were adjusted for age, sex, and refraction. The false-positive discovery rate method was applied to correct p-values for multiple comparisons. For all multivariable models, strength as assessed by standardized β, as well as the direction, of the associations are color-coded. Thus, positive (in red/warmer colors) and negative (in blue/cooler colors) associations are shaded based on the respective standardized β coefficients. The exact standardized β coefficients is given for all significant models with $p<0.05$. If the respective linear regression model did not show an overall significance (indicating that the standardized β is not valid for this association), no exact standardized β coefficients are depicted. Number of subjects included in all multivariate models for the respective marker of glucose homeostasis are provided.

Abbreviations are indicated in ESM Figure 3.

ESM Fig. 7

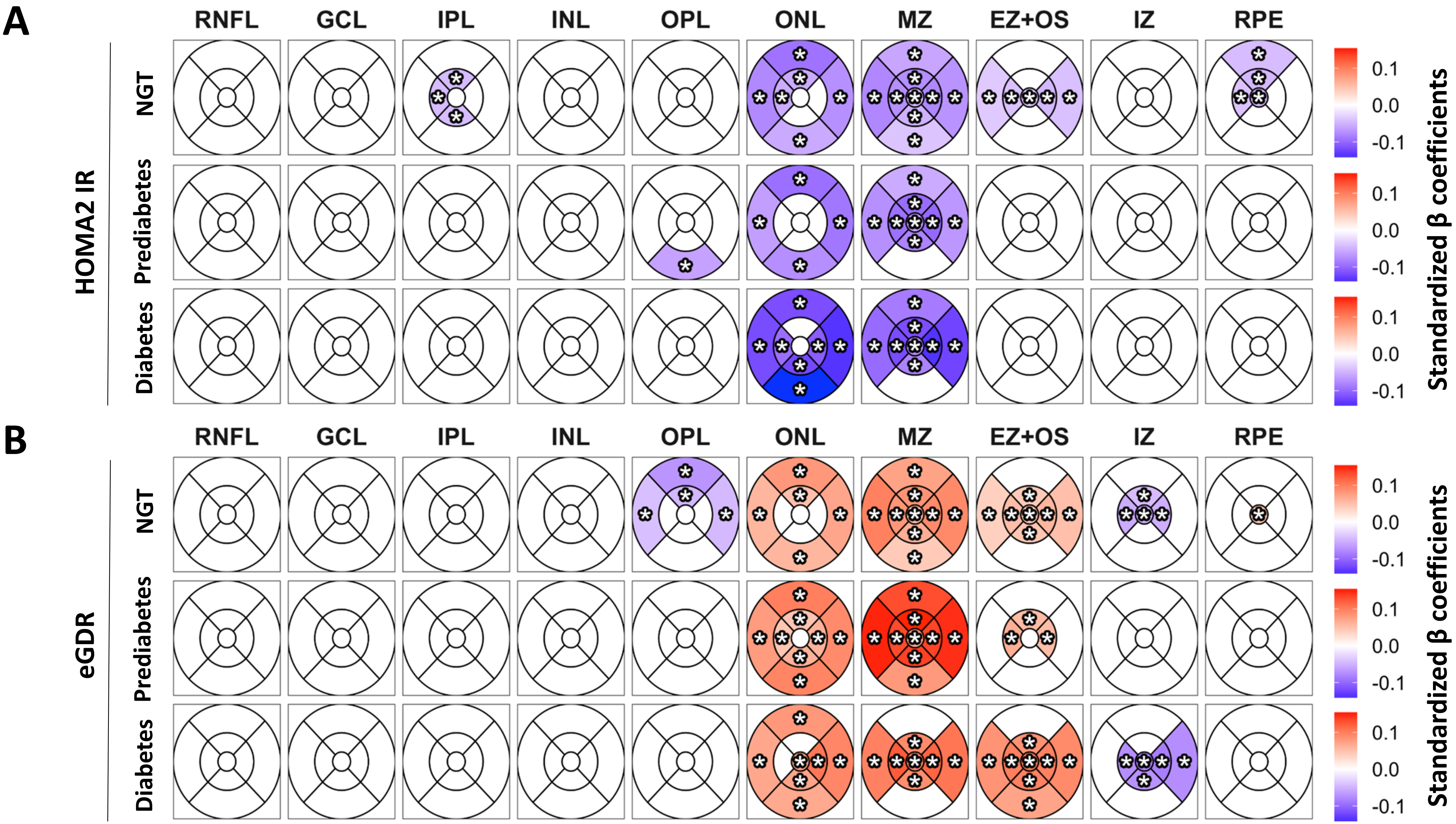
A
RNFL
GCL
IPL
INL
OPL
ONL
MZ
EZ+OS
IZ
RPE
HOMA2 IR
NGT
Prediabetes
Diabetes
0.1
0.0
-0.1
Standardized β coefficients
B
eGDR

ESM Figure 7

Subfield-specific associations between the retinal thickness of macular bands averaged within the commonly used Early Treatment Diabetic Retinopathy Study (ETDRS) subfields and HOMA2 IR (marker for insulin resistance, **panel A**) and eGDR (marker for insulin sensitivity, **panel B**) in subgroups of glucose homeostasis, i.e. normal glucose tolerance, prediabetes, and diabetes. Standardized β coefficients are color-coded depicting the strength and direction of the association. Thus, positive (in red/warmer colors) and negative (in blue/cooler colors) associations are shaded based on the respective standardized β coefficients. Asterisks denote statistical significant associations after adjustment for multiple comparisons by the false-positive discovery rate method. In all non-colored (i.e. white) subfields, no statistical significance was observed. Abbreviations are indicated in ESM Figures 3 and 4.

References

1. Korb CA, Kottler UB, Wolfram C, et al (2014) Prevalence of age-related macular degeneration in a large European cohort: Results from the population-based Gutenberg Health Study. Graefes Arch Clin Exp Ophthalmol 252(9):1403–1411. https://doi.org/10.1007/s00417-014-2591-9

2. van Leeuwen R, Klaver CCW, Vingerling JR, Hofman A, de Jong PTVM (2003) The Risk and Natural Course of Age-Related Maculopathy: Follow-up at 6½ Years in the Rotterdam Study. Arch Ophthalmol 121(4):519–526. https://doi.org/10.1001/archopht.121.4.519

3. Klaver CCW, Assink JJM, van Leeuwen R, et al (2001) Incidence and Progression Rates of Age-Related Maculopathy: The Rotterdam Study. Invest Ophthalmol Vis Sci 42(10):2237–2241

4. Loeffler M, Engel C, Ahnert P, et al (2015) The LIFE-Adult-Study: objectives and design of a population-based cohort study with 10,000 deeply phenotyped adults in Germany. BMC Public Health 15:691. https://doi.org/10.1186/s12889-015-1983-z

5. Inker LA, Schmid CH, Tighiouart H, et al (2012) Estimating Glomerular Filtration Rate from Serum Creatinine and Cystatin C. N Engl J Med 367(1):20–29. https://doi.org/10.1056/NEJMoa1114248

6. Kidney Disease: Improving Global Outcomes (KDIGO) CKD Work Group (2013) KDIGO 2012 Clinical Practice Guideline for the Evaluation and Management of Chronic Kidney Disease. Kidney Int Suppl 3(1):1–150. https://doi.org/10.1038/kisup.2012.64

7. Tönjes A, Kralisch S, Hoffmann A, et al (2019) Circulating Pro-Neurotensin in gestational diabetes mellitus. Nutr Metab Cardiovasc Dis 29(1):23–29. https://doi.org/10.1016/j.numecd.2018.09.011

8. American Diabetes Association (2021) 2. Classification and Diagnosis of Diabetes: Standards of Medical Care in Diabetes—2021. Diabetes Care 44(Supplement 1):S15–S33. https://doi.org/10.2337/dc21-S002

9. Craig CL, Marshall AL, Sjöström M, et al (2003) International Physical Activity Questionnaire: 12-Country Reliability and Validity. Med Sci Sports Exerc 35(8):1381. https://doi.org/10.1249/01.MSS.0000078924.61453.FB

10. von Hanno T, Lade AC, Mathiesen EB, Peto T, Njølstad I, Bertelsen G (2017) Macular thickness in healthy eyes of adults (N = 4508) and relation to sex, age and refraction: the Tromsø Eye Study (2007–2008). Acta Ophthalmol (Copenh) 95(3):262–269. https://doi.org/10.1111/aos.13337

11. Kass RE, Raftery AE (1995) Bayes Factors. J Am Stat Assoc 90(430):773–795. https://doi.org/10.2307/2291091

12. Madrigal-González J, Calatayud J, Ballesteros-Cánovas JA, et al (2020) Climate reverses directionality in the richness–abundance relationship across the World's main forest biomes. Nat Commun 11(1):5635. https://doi.org/10.1038/s41467-020-19460-y

13. Guerrero-Romero F, Simental-Mendía LE, González-Ortiz M, et al (2010) The Product of Triglycerides and Glucose, a Simple Measure of Insulin Sensitivity. Comparison with the Euglycemic-Hyperinsulinemic Clamp. J Clin Endocrinol Metab 95(7):3347–3351. https://doi.org/10.1210/jc.2010-0288

14. Epstein EJ, Osman JL, Cohen HW, Rajpathak SN, Lewis O, Crandall JP (2013) Use of the Estimated Glucose Disposal Rate as a Measure of Insulin Resistance in an Urban Multiethnic Population With Type 1 Diabetes. Diabetes Care 36(8):2280–2285. https://doi.org/10.2337/dc12-1693

15. Belfiore F, Iannello S, Volpicelli G (1998) Insulin Sensitivity Indices Calculated from Basal and OGTT-Induced Insulin, Glucose, and FFA Levels. Mol Genet Metab 63(2):134–141. https://doi.org/10.1006/mgme.1997.2658

16. Stumvoll M, Haeften TV, Fritsche A, Gerich J (2001) Oral Glucose Tolerance Test Indexes for Insulin Sensitivity and Secretion Based on Various Availabilities of Sampling Times. Diabetes Care 24(4):796–797. https://doi.org/10.2337/diacare.24.4.796

17. McAuley KA, Williams SM, Mann JI, et al (2001) Diagnosing Insulin Resistance in the General Population. Diabetes Care 24(3):460–464. https://doi.org/10.2337/diacare.24.3.460

Table Nine-Point Advised Protocol for OCT Study Terminology and Elements (APOSTEL) Checklist (Adapted from Cruz-Herranz et al., APOSTEL 2.0 recommendations for reporting quantitative optical coherence tomography studies. Neurology. 2021;97(2):68-79. PMID 33910937)

Item	Category	Recommendation
1	Study protocol	(1) Describe how many OCT operating sites and graders were included<br>Optical coherence tomography imaging was acquired at one operating site for the Leipzig Research Centre for Civilization Diseases LIFE-Adult Study; two experienced and clinically trained graders with consensus decision were employed.<br>(1) Report the timing of OCT compared to other measurements (same day, delayed)<br>All reported data of this manuscript was obtained on the same day.<br>(2) Describe the inclusion and exclusion criteria<br>B-scans with signal strength <20 dB were excluded from data analyses. The inclusion and exclusion process is depicted in Supplementary Figure S1. We sequentially excluded subjects with missing SD-OCT scans (excluded N = 931), as well as eyes with any clinically significant retinal findings in the macular and optic nerve head regions (N = 1,616). For this purpose, two independent, experienced, and clinically trained observers analyzed OCT scans and fundus images. In case of inter-observer differences, a consensus decision was reached to classify the participant's eye. Clinical and sub-clinical ophthalmic findings were graded based on current ophthalmological standards. Eyes with clinical disease of the posterior eye within macula or optic nerve regions were excluded from the current study: all cases of retinal detachment or retinal hole, retinal pigment epithelium detachment, edema, bleeding, vascular abnormalities (such as vascular occlusion, ischemia, retinal vascular tortuosity, aneurysm, neovascularization), any kind of scarring, atrophy, fundus with disseminated white areas, cotton-wool-spots, fibrosis if traction or puckering with foveal involvement was observed. The subject was also excluded if a tumor was present, or a staphyloma was detected. Within the macular region in specific, the following additional exclusion applied: age related macular degeneration (AMD) stages 2b, 3, 4a, and 4b, and maculopathy unrelated to AMD (stage 5), previously described by the Gutenberg Health Study [Korb CA et al., Graefes Arch Clin Exp Ophthalmol 2014] and based on the Rotterdam classification [van Leeuwen R et al., Arch Ophthalmol; Klaver CCW Invest Ophthalmol Vis Sci 2001]. Furthermore, subjects with missing diabetes diagnosis results (N = 69) were also excluded from data analyses, totaling to 2,616 excluded subjects (Supplementary Figure S1). For the remaining subjects (N = 7,384), one eye was randomly selected if both eyes of an included subject were reliable.
2	Acquisition device	For all OCT devices used, report data on:<br>(1) Manufacturer: Heidelberg Engineering, Heidelberg, Germany<br>(2) Model acquisition software: Heyex<br>(3) Version: 1<br>(4) Software version: 5.4.7.0<br>(5) Device type (time/spectral domain, swept-source, adaptive optics)[a] Spectralis (spectral domain)
3	Acquisition settings	Clearly describe the settings in which OCT scans were obtained:<br>(1) Pupils dilated before examination (y/n) no<br>(2) Number of operators and devices[b] For the Leipzig Research Centre for Civilization Diseases LIFE-Adult Study one acquisition device is employed where three epidemiologically trained study nurses obtain ophthalmological data and images by strictly adhering to the Standard Operating Procedure.
4	Scanning protocol	Clearly describe the scanning protocol, including:<br>(1) Type of scan (circular, volume, star, line, other)<br>The scanning protocol consists of a fovea-centred volume scan, a volume scan and a cpRNFLT ring scan both centred on the optic nerve head, and an enhanced-depth imaging line scan.<br>(2) Location (area of interest, macula, optic nerve head (ONH), papillomacular bundle, other)<br>Both macula and optic nerve head (ONH) regions were scanned.<br>(3) Scan parameters (with eye tracking) Volume scan: 20° fovea centred and 15° optic disc centred, number of B-scans: 97 (fovea centred), 49 (ONH centred), horizontal alignment of B-scans, 512 A-scans per B-scans, X: 512 pixels (11.58μm/pixel) and Z: 496 pixels (3.87 μm/pixel).<br>Ring scan: 12° diameter, 768 A-scans per B-scan, manual placement of ring centred on ONH, depth resolution: X: 768 pixels (14.55μm/pixel) Z: 496 pixels (3.87 μm/pixel). Line scan: horizontal (0° angle), location, number of A-scans, depth resolution: enhanced depth imaging , X: 1536 pixels (5.63μm/pixel) Z: 496 pixels (3.87 μm/pixel).
5	Funduscopic imaging	(1) Report other imaging modalities used in addition to OCT (funduscopy, confocal scanning laser ophthalmoscope, retinal angiography, autofluorescence imaging, etc.)<br>Non-mydriatic fundus photography images (Nidek AFC-230), 45° macula centred, were obtained.<br>SLO Fundus image is was obtained.<br>(2) Describe acquisition protocol, including:<br>Excitation wavelength NA<br>Filter sets NA<br>Number of frames averaged (if applicable): ART mode: Volume scan: 10 A-scans averaged, ring scan: 100 A-scans averaged, Line-scan: 100 A-scans averaged, SLO: 100 images averaged<br>Report device specific features when utilized (e.g., enhanced depth imaging, swept-source OCT, adaptive optics)[a]
6	Postacquisition data selection	Describe image selection process, including:<br>(1) Quality control criteria<br>(2) Postacquisition discard (number and criteria)<br>(3) Eye selection strategy (if applicable)
7	Postacquisition analysis	Describe all postacquisition steps:<br>(1) Software used for processing scans and segmentation (may be different from acquisition software): Retinal bands were automatically segmented by HEYEX software version 6.16.8 (Heidelberg Engineering, Heidelberg, Germany) yielding 10 different retinal bands.<br>(2) Which individual retinal layers were segmented/included: segmented retinals bands were: RNFL, GCL, IPL, INL, OPL, ONL, which includes thickness of external limiting membrane (ELM) as this layer is segmented by Heyex software at the inferior border of the bright band (ELM); MZ; EZ and OS combined (named EZ+OS here) as

		segmentation by Heyex software of this section consists of a bright (EZ) and a dark (OS) band, IZ, and RPE.<br>(3) Method of segmentation (automated, semi-automated, or manual): automated<br>(4) How potential bias was addressed in the case of manual segmentation or manual correction of automated segmentation errors (masking)[a] NA<br>(5) Grid used for data extraction (size, shape, selected sections): ETDRS grid consisting of a 6mm circle, a 3mm circle and a 1mm circle with 9 sectors and global thickness.<br>(6) Pixel to millimeter ratio if images are exported (caliper need)[a]: See point 4(2) above
8	Nomenclature and abbreviations	Define:<br>(1) Anatomical structures analyzed: RNFL, GCL, IPL, INL, OPL, ONL, which includes thickness of external limiting membrane (ELM) as this layer is segmented by Heyex software at the inferior border of the bright band (ELM); MZ; EZ and OS combined (named EZ+OS here) as segmentation by Heyex software of this section consists of a bright (EZ) and a dark (OS) band, IZ, and RPE.<br>(2) Units of provided measurements (e.g., volume or thickness): thickness in μm, and volume in mm^2<br>(3) Report the number of eyes presenting additional retinal pathology; describe qualitative retinal changes and report exact methodology of quantification[a] See study protocol method section above, in brief:clinical significant alterations were excluded for the present analysis.
9	Statistical approach	Describe:<br>(1) Statistical models used for the analyses of OCT data: see method section for this analysis<br>(2) Whether data were analyzed by eye or by patient: data was always analysed per eye.

Abbreviation: OCT = optical coherence tomography.
The modified APOSTEL checklist containing 9 important items when reporting quantitative OCT studies.
[a] Changes made to the original APOSTEL recommendations checklist.
[b] Room light conditions were removed.
